# Ensemble Ground State of a Many-Electron System with Fractional Electron Number and Spin: Piecewise-Linearity and Flat-Plane Condition Generalized

**DOI:** 10.1021/acs.jpclett.3c03509

**Published:** 2024-02-22

**Authors:** Yuli Goshen, Eli Kraisler

**Affiliations:** Fritz Haber Research Center for Molecular Dynamics and Institute of Chemistry, The Hebrew University of Jerusalem, 9091401 Jerusalem, Israel

## Abstract

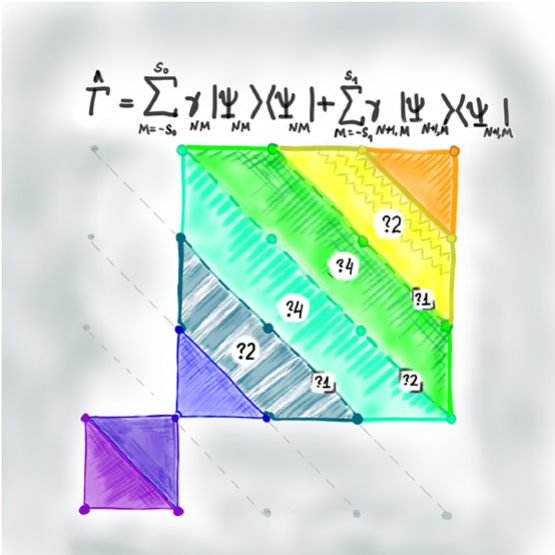

Description of many-electron systems with a fractional electron number (*N*_tot_) and fractional spin (*M*_tot_) is of great importance in physical chemistry, solid-state physics, and materials science. In this Letter, we provide an exact description of the zero-temperature ensemble ground state of a general, finite, many-electron system and characterize the dependence of the energy and the spin-densities on both *N*_tot_ and *M*_tot_, when the total spin is at its equilibrium value. We generalize the piecewise-linearity principle and the flat-plane condition and determine which pure states contribute to the ground-state ensemble. We find a new derivative discontinuity, which manifests for spin variation at a constant *N*_tot_, as a jump in the Kohn–Sham potential. We identify a previously unknown degeneracy of the ground state, such that the total energy and density are unique, but the spin-densities are not. Our findings serve as a basis for development of advanced approximations in density functional theory and other many-electron methods.

Modeling of materials and chemical processes from first-principles crucially depends on efficient, accurate, yet approximate methods (see, for example, refs (1−6)). The quality of a given approximation can be assessed not only versus the experiment but also by comparison to known exact properties of many-electron systems; in the context of density functional theory (DFT) (see, for example, refs (7−9)), one such exact property is the piecewise-linear behavior of the energy versus electron number:^10^ when varying the number of electrons in the system, *N*_tot_, in a continuous manner, allowing both integer and fractional values, the energy *E*(*N*_tot_) is linear between any two integer values of *N*_tot_. As *N*_tot_ surpasses an integer, the slope of *E*(*N*_tot_) may abruptly change. *Piecewise linearity* exists also for the electron density *n*(**r**) and any property that is an expectation value of an operator.

Spin, which is a fundamental property of an electron, has to be taken into account in many-electron systems, both when magnetic fields are present, but also in their absence.^11−13^ Occurrence of fractional *z*-projection of the total spin, *M*_tot_, and the dependence of the energy on *M*_tot_ have been extensively studied in the literature.^14−22^ In ref (15) it was shown that the exact total energy must be constant for all *M*_tot_ ∈ [−*S*_min_, *S*_min_] (where *S*_min_ is the *equilibrium* value of the *total* spin, *S*, i.e., that value of *S* for which the system has the lowest energy). In ref (16) the *flat-plane condition* was developed, unifying piecewise-linearity^10^ and the constancy condition,^15^ which are both completely general principles and apply to any many-electron approach. Focusing on the H atom, ref (16) described its exact total energy, while continuously varying *N*_tot_ from 0 to 2 and *M*_tot_ accordingly, while . (Hartree atomic units are used throughout.) The exact energy graph of H in the *N*_*↑*_ – *N*_*↓*_ plane (where  and ) is comprised of two triangles whose vertices lie at the points of integer *N*_*↑*_ and *N*_*↓*_. Approximate exchange–correlation (xc) functionals may strongly violate this condition, leading to a delocalization error and static correlation error.

These developments were followed by numerous studies (see, for example, refs (23−28) and references therein) which focused on (a) the constancy condition,^15^ examined by changing *M*_tot_ between – *S*_min_ and + *S*_min_ while keeping *N*_tot_ constant and integer, or (b) varying both *N*_tot_ and *M*_tot_ for systems with . In addition, refs (14, 17, 20, and 22) analyzed the flat-plane behavior of the energy for general *N*_*↑*_ and *N*_*↓*_, suggesting formulas describing the energy profile. They assumed that the total energy (and analogously other properties) for any fractional *N*_tot_ and *M*_tot_ is determined by the energies of three out of the four states with nearby integer numbers of *↑* and *↓* electrons. The overall energy graph is, therefore, a collection of triangles. Moreover, refs (29 and 18) raised the possibility of a discontinuity in *E* versus *M*_tot_, which stems from the nonuniqueness of the external magnetic field in spin-DFT.

This work goes beyond previous results and rigorously describes the exact ground state of a general, finite, many-electron system with an arbitrary fractional electron number, *N*_tot_, and a simultaneously fractional *z*-projection of the spin *M*_tot_, as long as the total spin, *S*, is at its equilibrium value, *S* = *S*_min_; *S*_min_ itself can be of any value. Such a ground state has to be described by an ensemble, attained by minimizing the total energy while constraining *N*_tot_ and *M*_tot_.

Our findings generalize the piecewise-linearity principle^10^ and the flat-plane condition.^16^ We find which pure ground states contribute to the ensemble and which do not. The contributing pure states are not necessarily among the four neighboring states to the point (*N*_tot_, *M*_tot_), contrary to previous findings. Notably, the energy graph consists not only of triangles but also of trapezoids. Furthermore, we identify a new type of a derivative discontinuity, which manifests in the case of spin variation, as a jump in the Kohn–Sham (KS) potential and compare it to the discontinuities defined in ref (12). Surprisingly, we reveal a degeneracy in the ensemble ground state, where the energy and the total density are determined uniquely but the spin-densities are not. Our findings provide new exact properties of many-electron systems, which are useful for development of advanced approximations in DFT and in other many-electron methods.

We start our derivation by considering *an ensemble* ground state Λ̂ of a system with possibly fractional *N*_tot_ and fractional *M*_tot_, where no magnetic fields are present. The (nonrelativistic) Hamiltonian is given by , where *T̂* is the kinetic energy operator, *V̂* is a multiplicative external potential operator, and *Ŵ* is the electron–electron repulsion operator. Subsequently, Λ̂ minimizes the expectation value of the energy, , under the constraints
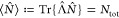
1
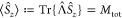
2and
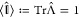
3Here,  is the electron number operator and  is the operator for the *z*-projection of the total spin.

Consider now |Ψ_*N*,*S*,*M*_⟩, the lowest-energy eigenstate of the operators , with eigenvalues *N*, *S*(*S* + 1), and *M*, respectively. *N* is a non-negative integer, *M* and *S* are integers (for even *N*) or half-integers (for odd *N*), and – *S* ⩽ *M* ⩽ *S*. For a given value of *N*, there exists one such value of *S* that minimizes the energy; we denote it *S*_min_. Subsequently, there exists a multiplet of (2*S*_min_ + 1) states, all with the same energy (in absence of a magnetic field), and with *M* = −*S*_min_, ..., *S*_min_. We assume that for all other values of *S* (each one with its own multiplet of states), the energy is strictly higher. We further assume, for simplicity, that for specified *N*, *S*, and *M*, the pure ground state is not degenerate any further.

In the following we denote |Ψ_*N*,*M*_⟩ as the lowest-energy eigenstate of  and , with eigenvalues *N* and *M*, respectively. For *M* ∈ [−*S*_min_, *S*_min_], we have, of course, |Ψ_*N*,*M*_⟩ = |Ψ_*N*,*S*_min_(*N*),*M*_⟩; varying *M* does not affect the energy . For other values of *M*, the ground state energy is higher. The energy  as a function of *S* and *M* is as illustrated in Figure 1. Moreover, as long as *M* ∈ [−*S*_min_(*N*), *S*_min_(*N*)], the density  does not depend on *M* (see the Supporting Information (SI) for details).

**Figure 1:**
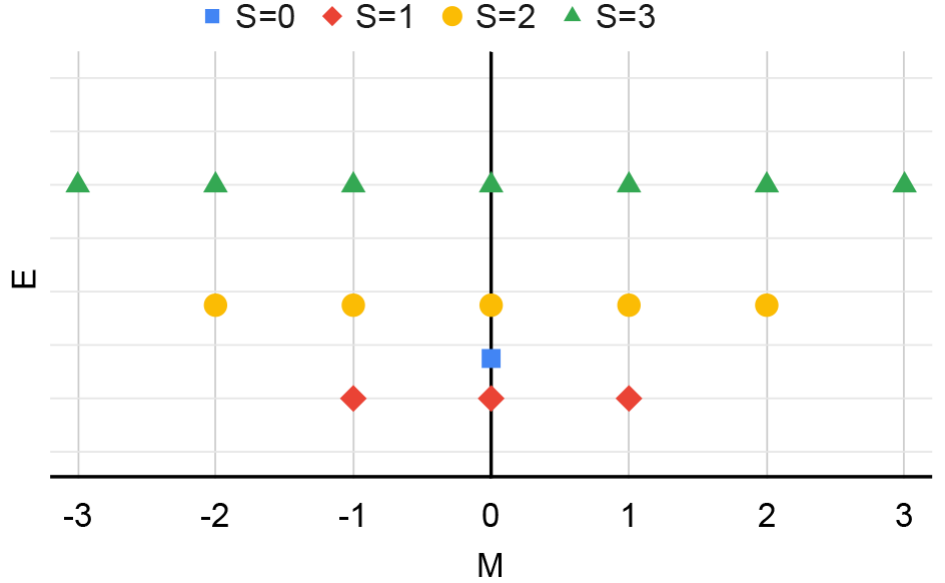
Illustration of the energy  versus *M*, at constant *N*, for various values of *S* (see legend). In this case, *S*_min_ = 1.

For general, possibly fractional, values of *N*_tot_ and *M*_tot_, the ground state Λ̂ is expressed as Λ̂ =  where λ_*N*,*M*_ ∈ [0, 1]. Here and below the sum over *N* includes all integers for which the *N*-electron system is bound, and the sum over *M* includes all possible (integer or half-integer) values of *M*, for a given value of *N*: . We use here the fact that , , , and  are commuting operators and therefore their eigenstates, |Ψ_*N*,*S*,*M*,*E*_⟩, form a complete basis. Among these, only |Ψ_*N*,*M*_⟩ are necessary to describe the ground state Λ̂, as they have the lowest energy for a given *N* and *M*. We note in passing that more general ensembles, which include also off-diagonal terms of the form |Ψ_*N*_*a*_,*M*_*a*__⟩⟨Ψ_*N*_*b*_,*M*_*b*__|, introduce additional degeneracy to the ground state, but the energy and the spin-densities *n*_ens_^σ^(**r**) are unaffected by this extension. In the following, we denote *N*_tot_ = *N*_0_ + α, where *N*_0_ is the integer part and α ∈ [0, 1) is the fractional part of *N*_tot_. The equilibrium spin values for *N*_0_ and *N*_0_ + 1 electrons are, respectively, *S*_0_ ≔ *S*_min_(*N*_0_) and *S*_1_ ≔ *S*_min_(*N*_0_ + 1).

We now consider the important case of
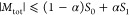
4and prove that *any* ensemble state, which consists of only |Ψ_*N*_0_,-*S*_0__⟩, ···, |Ψ_*N*_0_,*S*_0__⟩ and |Ψ_*N*_0_+1,-*S*_1__⟩, ···, |Ψ_*N*_0_+1,*S*_1__⟩, and satisfies constraints 1–3, is *a ground state* of the system.

By explicitly writing the aforementioned ensemble state as
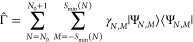
5and making use of eqs 1 and 3 (for Γ̂), we see that  and . It then follows that the energy of this ensemble state is , where *E*(*N*) ≔ min_*M*_*E*(*N*, *M*) = *E*(*N*, *S*_min_(*N*)) is the ground state energy for a given *N*. Notably, there is no dependence on the *z*-projection of the spin; constraint 2 was not used. Now we show that  is indeed the ground state energy.

First, from the piecewise-linearity principle of ref (10), it follows that min_Ξ̂→*N*_tot__ = (1 − α)*E*(*N*_0_) + α*E*(*N*_0_ + 1), namely, that the ground state energy for any system with *N*_tot_ electrons, represented by a state Ξ̂, is linear in *N*, between *N*_0_ and *N*_0_ + 1. Second, note that *E*_ens_(*N*_tot_,*M*_tot_) ≔ min_Ξ̂→*N*_tot_,*M*_tot__ ⩾ min_Ξ̂→*N*_tot__, merely stating the ensemble ground-state energy for given *N*_tot_ and *M*_tot_ is greater or equal to the ensemble ground-state energy given only *N*_tot_, because adding an additional constraint in a minimization yields a result larger or equal the original one. Third, since Γ̂ satisfies eqs 1–3, meaning that Γ̂ is an ensemble that yields *N*_tot_ and *M*_tot_, . Combining all the above, we find that
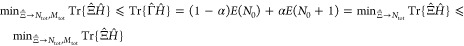
6This inequality holds only if all its terms equal each other, meaning that , i.e., Γ̂ is indeed *a* ground state.

Next, to show that *every* ground-state is of the form of Γ̂ (eq 5), still within Region 4, we assume, by way of contradiction, that this is false for a ground state Λ̂, which is explicitly written as . Next, we define an auxiliary quantity
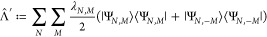
7to deduce properties of Λ̂. The sum on *M* in eq 7 runs over both positive and negative values, as before.  satisfies eqs 1 and 3, , and  =  = (1 − α)*E*(*N*_0_) + α*E*(*N*_0_ + 1). Therefore,  is itself a ground state of *N*_tot_ electrons and spin 0.

To find the coefficients λ_*N*,*M*_ that bring the energy of  to a minimum, while retaining its expectation values of , we notice that, *for each value of N* (in the external sum of eq 7), the energy is minimized by setting λ_*N*,*M*_ = 0 for all *M* > *S*_min_(*N*) and *M* < −*S*_min_(*N*). Then,  =  +  and  = ∑_*N*_*l*_*N*_*E*(*N*) where *l*_*N*_ =  λ_*N*,*M*_. This expression for the ensemble energy is familiar from ref (10). Using the convexity conjecture of the energy,^3,19,30^ we state that this energy is minimized by
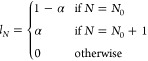
8still under the aforementioned constraints for . This means that the only nonzero coefficients of Λ̂ are  and , in contradiction to the original assumption.

This concludes our proof: the ground state of a system with *N*_tot_ electrons with a total spin *M*_tot_ is described *only* by the ensemble ground state Γ̂ of eq 5. It consists of 2(*S*_0_ + *S*_1_ + 1) pure states, at most. The coefficients γ_*N*,*M*_ ∈ [0, 1] have to be determined from Constraints 1–3, which can be expressed as
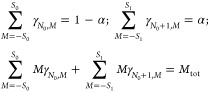
9

Several important consequences follow from the fact that Γ̂ of eq 5 is a ground state.

*1. Total Energy*. Within Region 4, the total energy
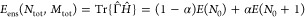
10is piecewise-linear with respect to α and does not depend on the *z*-projection of the spin, *M*_tot_. The graph of *E*_ens_(*N*_*↑*_, *N*_*↓*_) forms a series of triangles and trapezoids, as presented in Figure 2. For the H atom, with the number of electrons in each spin channel varying from 0 to 1, this result is precisely the flat-plane condition as described in ref (16). However, for larger *N*_tot_ and particularly for , e.g., the C atom of Figure 2, the behavior of *E*_ens_ is richer. Furthermore, since *E*_ens_ does not depend on the spin, the slope in Figure 2 along the lines of constant *N*_tot_ is (∂*E*_ens_/∂*M*_tot_)_*N*_tot__ = 0. This result is in agreement with ref (15).

**Figure 2:**
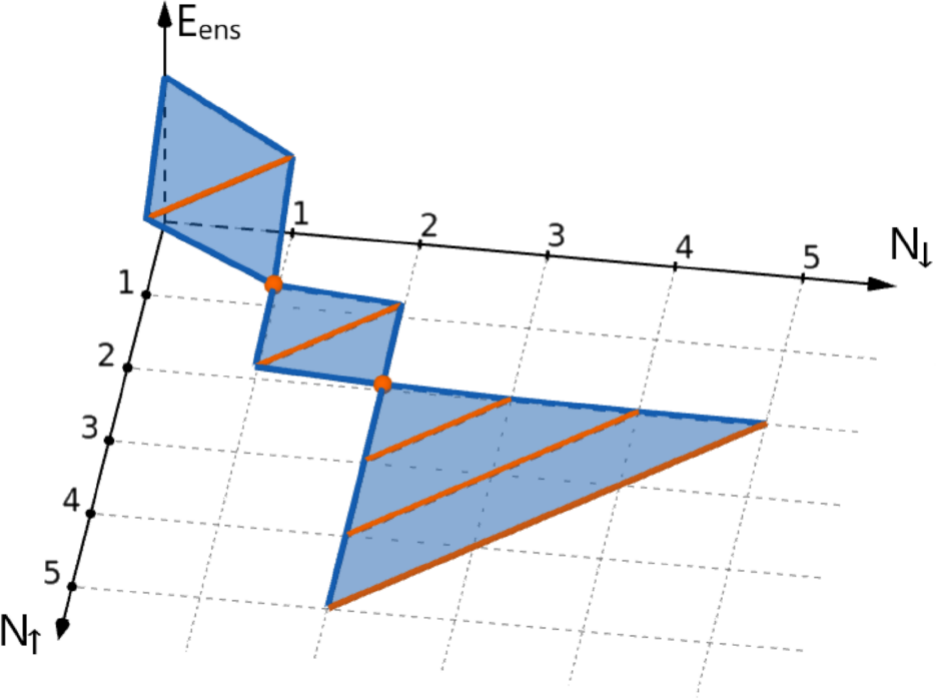
Ground-state energy *E*_ens_(*N*_*↑*_, *N*_*↓*_) for the C atom, within the region defined in eq 4, and for 0 ⩽ *N*_tot_ ⩽ 7. The slope is zero along the orange line segments, which correspond to constant and integer *N*_tot_, and changes discontinuously in directions perpendicular to these lines.

*2. Frontier Eigenvalues and Derivative Discontinuity*. We denote *M*_*B*_ = (1 – α)*S*_0_ + *αS*_1_, the highest value of *M*_tot_ (for a given *N*_tot_), at the *boundary* of Region 4, and consider |*M*_tot_| < *M*_*B*_. In this case, (∂*E*_ens_/∂*N*_↑_)_*N*_↓__ = (∂*E*_ens_/∂*N*_↓_)_*N*_↑__ = (∂*E*_ens_/∂*N*_tot_)_*M*_tot__, because (∂*E*_ens_/∂*M*_tot_)_*N*_tot__ = 0. Hence, by Janak’s theorem,^31^ the highest occupied (ho) KS energy eigenvalues are the same for both spin channels, constant strictly within each trapezoid/triangle as in Figure 2, and equal (the negative of) the corresponding ionization potential: ε_ho_^↑^ = ε_ho_^↓^ = *E*(*N*_0_+1) – *E*(*N*_0_).

However, at and beyond the boundary of Region 4, the value of ε_ho_^σ^ may differ from *E*(*N*_0_ + 1) – *E*(*N*_0_), as it is determined by the slope of the energy just outside and adjacent to the boundary. Whereas the full discussion of the energy profile outside Region 4 is beyond the scope of this work, we note that just outside the boundary the energy is a plane determined by three pure-state energy values, one of which is outside Region 4. Therefore, a change in the energy slope is expected as we cross the boundary *M*_tot_ = *M*_*B*_ of Region 4. Specifically, for *spin migration* (variation of *M*_tot_ at constant *N*_tot_), we predict a *derivative discontinuity* for the KS energy levels, which is manifested in a uniform jump in the KS potential *v*_KS_^σ^(**r**) at *M*_tot_ = *M*_*B*_ [a similar logic applies for the boundary *M*_tot_ = −*M*_*B*_].

We define the *spin-migration derivative discontinuity* around *M*_tot_ = *M*_*B*_ as
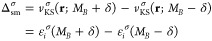
11where δ → 0^+^, *N*_tot_ is kept constant, and *i* refers to any of the KS energy levels. In the following, quantities evaluated at *M*_*B*_ + δ are denoted with a prime (e.g., ) and those evaluated within Region 4 are without a prime.

As an illustration, choose  and consider a common example, where immediately outside Region 4, for *M*_tot_ > *M*_*B*_, the ensemble ground state consists of |Ψ_*N*_0_,*S*_0__⟩, |Ψ_*N*_0_,*S*_0_+1_⟩, and |Ψ_*N*_0_+1,*S*_1__⟩. Then, the ensemble energy there is *E*_ens_^′^(*N*_tot_, *M*_tot_) = *KN*_tot_ + *JM*_tot_ + *C*, where *J* = *E*(*N*_0_, *S*_0_ + 1) – *E*(*N*_0_, *S*_0_) – a spin-flip energy, and *K* = (*E*(*N*_0_ + 1) – *E*(*N*_0_)) – (*S*_1_ – *S*_0_)*J*. Therefore, the slope changes around *M*_*B*_ equal
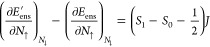
12
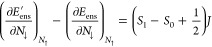
13Notably, the *↑*-slope is continuous for  and the *↓*-slope is continuous for .

Specifically for , we obtain a discontinuity only in the *↓*-channel:
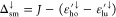
14We note that as *M*_tot_ surpasses *M*_*B*_, *N*_*↓*_ crosses an integer, while *N*_*↑*_ does not; the *↓*-ho orbital within Region 4 is the *↓*-lu orbital immediately outside it. The term in parentheses in eq 14 is (the negative of) the KS gap immediately outside Region 4. It equals ε_ho-1_^↓^–ε_ho_^↓^ in terms of quantities inside Region 4.

In the uncommon case of , we expect discontinuities in both spin channels, while neither *N*_*↑*_ nor *N*_*↓*_ cross an integer.

As to the value of the ho level at the boundary itself, it is determined by recalling that all of the derivatives with respect to *N*_σ_ are taken from below. Then, from geometric considerations in the *N*_*↑*_ – *N*_*↓*_ plane, it follows that ε_ho_^↑^(*M*_*B*_) = *E*(*N*_0_ + 1) – *E*(*N*_0_) if  and ε_ho_^↓^(*M*_*B*_) = *E*(*N*_0_ + 1) – *E*(*N*_0_) if . In other words, in the above cases, the ho level at the boundary has the same value as strictly within (4). Otherwise, at the boundary, it has the value immediately outside (4).

Δ_sm_^σ^ is related to the xc correction to the spin stiffness defined in ref (12), for the case of integer *N*_tot_: if immediately outside Region 4 the ensemble ground state consists of |Ψ_*N*_0_,*S*_0__⟩ and |Ψ_*N*_0_,*S*_0_+1_⟩, the two quantities coincide. However, in the general case they differ, as ref (12) expressly makes use of excited-state spin ensembles, following ref (32), and here we treat ground-state ensembles.

*3. Nonuniqueness and Degeneracy*. Remarkably, the coefficients γ_*N*,*M*_ of eq 5 are confined by only *three* constraints, eq 9, whereas the number of coefficients is 2(*S*_0_ + *S*_1_ + 1). This means that the ground state Γ̂ may not be uniquely defined. Strictly within Region 4 (i.e., |*M*_tot_| < *M*_*B*_) and , all 2(*S*_0_ + *S*_1_ + 1) coefficients may be nonzero. Then, since there are 3 constraints (eq 9), the degeneracy of the ground-state is (2*S*_0_ + 2*S*_1_ – 1)-fold. Similarly, if  and |*M*_tot_| < *S*_0_, then we are left with 2*S*_0_ + 1 coefficients and 2 constraints; hence, the degeneracy is (2*S*_0_ – 1)-fold. We discuss this nonuniqueness here in detail, starting with the cases where Γ̂ is defined uniquely.

*Case a*. If *S*_0_ = 0 and  (e.g., adding an electron to H^+^ toward H, while varying *M*_tot_), we are left with three uniquely determined γ_*N*,*M*_’s: , , and . Similarly, if  and *S*_1_ = 0 (e.g., adding an electron to H toward H^–^, while varying *M*_tot_), we also have three uniquely defined coefficients: , , and .

*Case b*. At the boundary of Region 4, where the spin is at its maximal value, *M*_tot_ = *M*_*B*_, we find that the expression for *M*_tot_, , reaches its maximum (under the aforementioned constraints on γ_*N*,*M*_’s) only when ,  and all the other γ_*N*,*M*_’s vanish; the ground state is determined uniquely. If, for example, , this corresponds to the common scenario of *ionization* (via the up spin channel): fractionally varying *N*_*↑*_ while *N*_*↓*_ is a constant integer. A unique solution is obtained also when *M*_tot_ reaches its minimal value, i.e., for *M*_tot_ = −*M*_*B*_.

*Case c*. For the scenario of *spin migration*, when *N*_tot_ = *N*_0_ = const. and *M*_tot_ varies between −*S*_0_ and *S*_0_, the ground state is *not* defined uniquely, for *S*_0_ ⩾ 1. From eq 9 we see that  and therefore , for all *M*. We are then left with 2*S*_0_ + 1 coefficients.

In particular, for *S*_0_ = 1 (e.g., spin migration for the C atom), the three coefficients , , and  can be expressed as , , and , where *x* remains *undetermined*. To understand the meaning of the above ambiguity in the ground state, focus on the case of *M*_tot_ = 0. Then,  =  +  + meaning that to get the average , the system must have an equal probability of  to be in the *M* = 1 and in the *M* = −1 states and a complementary probability of (1 – *x*) to be in the state with M = 0. Here we see a clear distinction of constraining the *average* spin of the system being 0 versus requiring each and every replica in a macroscopic statistical ensemble to have a spin of 0. The latter is obtained by demanding that the ground state of the system is an eigenstate of , with eigenvalue 0 (which is equivalent here to setting *x* = 0).

Surprisingly, despite the ambiguity in Γ̂, in this Case the density, as well as the spin-densities, is determined unambiguously and does not depend on *x*. Since the pure-state densities *n*_*N*,*M*_(**r**) do not depend on *M* (see the SI for details), *n*_ens_(**r**) = *n*_*N*_0__(**r**). For the spin-densities, , where δ_*↑*_ = 1 and δ_*↓*_ = −1. The spin distribution, defined by *Q*(**r**) ≔ *n*_*↑*_(**r**) – *n*_*↓*_(**r**), is expressed for our ensemble as
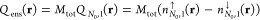
15being linear in *M*_tot_, and independent of *x*. Here we used the fact that for any pure state |Ψ_*N*,*M*_⟩, *n*_*N*,-*M*_^↑^(**r**) = *n*_*N*,*M*_^↓^(**r**).

*Case d*. For spin migration with  (e.g., for the N atom), even the spin-densities are *not* determined uniquely anymore. Here we have four coefficients (dropping the index *N*_0_ for brevity): γ_3/2_ = *x* + (*M*_tot_ − *y*), γ_1/2_ =  (1 − *x*) + *y*, γ_−1/2_ = (1 − *x*) − *y*, and γ_−3/2_ = *x* − (*M*_tot_ − *y*), with two undetermined parameters, *x* and *y*. Whereas the total energy and total density are determined unambiguously, the spin-densities linearly depend on *y* and are independent of *x*. The ensemble spin distribution
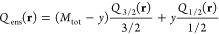
16is linear in *y*.

*Case e*. For a fractional *N*_tot_ = *N*_0_ + α,  and *S*_1_ = 1 (e.g., adding an electron to C^+^ toward C, while varying *M*_tot_), we end up with five coefficients, which depend on two parameters, *x* and *y*: γ_*N*0,1/2_ = (1 − α) + *y*, γ_*N*_0_,–1/2_ = (1 − α) − *y*, γ_*N*_0_+1,1_ =(α − *x*) + (*M*_tot_ − *y*), γ_*N*_0_+1,0_ = *x*, and γ_*N*_0_+1,−1_ = (α − *x*) − (*M*_tot_ − *y*). The total ensemble density *n*_ens_(**r**) = (1 – α)*n*_*N*_0__(**r**) + *αn*_*N*_0_+1_(**r**) is piecewise-linear in α and remains fully determined, whereas the spin-densities are not. The ensemble spin distribution is
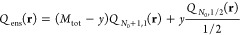
17being linear in *y*, but independent of α (in full contrast to *n*_ens_(**r**)).

Cases d and e clearly show that for a given electron number and spin we have a set of degenerate ground states Γ̂(*x*,*y*), with the *same* total density, but with different spin-densities, *n*_ens_^↑^(**r**) and *n*_ens_^↓^(**r**). Notably, even in the spin-unpolarized case *M*_tot_ = 0, the spin-densities are not determined and are not necessarily equal to each other.

*4. Removing Nonuniqueness*. To further explore and interpret the surprising result of nonuniqueness at the ground state, we offer two ways for its full or partial removal.

First, to determine the ground state unambiguously, one can introduce additional constraints to complement 1–3. For example, we can set , , and/or higher moments of . In the SI we analyze how many such moments are sufficient to determine  and/or *Q*_ens_(**r**), in the general case. Specifically, for Case c above, , i.e., setting  fully determines Γ̂.

Alternatively, one can require the standard deviation Δ*S*_*z*_ ≔  to be minimal. This requirement sets the ground state *uniquely*; a mathematical proof of this statement is provided in the SI. In particular, in Case c the deviation is Δ*S*_*z*_ = . For all coefficients γ_*N*,*M*_ to remain within [0, 1], *x* is confined to [|*M*_tot_|, 1], and therefore . Notably, for minimal Δ*S*_*z*_ we are left with only two nonzero coefficients: For *M*_tot_ ∈ [0, 1],  and ; for *M*_tot_ ∈ [−1, 0],  and . Thus, as *M*_tot_ goes from 1 to −1, we observe a gradual transition between |Ψ_*N*_0_,1_⟩⟨Ψ_*N*_0_,1_| to |Ψ_*N*_0_,0_⟩⟨Ψ_*N*_0_,0_| and then to |Ψ_*N*_0_,-1_⟩⟨Ψ_*N*_0_,-1_|, with two adjacent pure states involved at each point.

In Case d, , as well as all even moments of  equal , being linear in *x* and independent of *y*. Conversely, all the odd moments  are linear in *y* and independent of *x*. Therefore, it is sufficient to set  and , or to minimize Δ*S*_*z*_ (see the SI for the technical details). In the latter case, we obtain a gradual transition from |Ψ_*N*_0_,3/2_⟩⟨Ψ_*N*_0_,3/2_| to |Ψ_*N*_0_,1/2_⟩⟨Ψ_*N*_0_,1/2_| to |Ψ_*N*_0_,–1/2_⟩⟨Ψ_*N*_0_,–1/2_| to |Ψ_*N*_0_,–3/2_⟩⟨Ψ_*N*_0_,–3/2_|, with two adjacent pure states involved at each point. The spin distribution is unambiguously determined and is piecewise-linear in *M*_tot_:
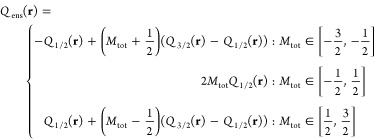
18

Second, to remove nonuniqueness of the ground state it is natural to introduce a weak magnetic field. (The magnetic field being weak means that μ_B_*B*_0_ is smaller than any energy difference in the problem; *f*(**r**) is bounded. Therefore, we refer only to terms that are first order in **B**.) Surprisingly, while lifting the degeneracy between pure states |Ψ_*N*,*M*_⟩ with different *M*, a magnetic field does not fully remove the nonuniqueness.

Consider an inhomogeneous magnetic field . Then, the pure-state energies become  = *E*(*N*) +  = , where *F*_*N*,*M*_ =  for *M* ≠ 0 and 0 otherwise. (In our analysis we disregard, for simplicity, the magnetic term **A**·**p̂**, where **A** is the electromagnetic vector-potential and **p̂** is the momentum operator. [For homogeneous magnetic fields, this term boils down to **B**·**L̂**.] Treatment of this term changes the value of the energy *E*(*N*), but it is independent of *M*, which is our main focus.)

Applying this magnetic field in Case d, the ensemble energy becomes *Ẽ*_ens_(*N*_0_, *M*_tot_) = *E*(*N*_0_) + μ_B_*B*_0_*M*_tot_*F*_*N*_0_,3/2_ + μ_B_*B*_0_ (*F*_*N*_0_,1/2_ − *F*_*N*_0_,3/2_)*y*, being linear in *y*. One can then minimize  with respect to *y*: If, say, for a given system and field profile , then for *B*_0_ > 0, we seek for the lowest possible *y*, and for *B*_0_ < 0, for the highest. The range of *y* values is confined by the requirement γ_*N*,*M*_ ∈ [0, 1]. In this case, it results in *x* ∈ [0, 1], , and . Consequently, the lowest possible *y* is , for  and , for . Fortunately, minimizing *y* in this Case also uniquely sets *x*, and therefore, the ground state is determined uniquely. As in the case of minimal Δ*S*_*z*_, here, we are also left with only two pure states involved in the ensemble at each point. Now, however, we perform a gradual transition from |Ψ_*N*_0_,3/2_⟩⟨Ψ_*N*_0_,3/2_| to |Ψ_*N*_0_,–1/2_⟩⟨Ψ_*N*_0_,–1/2_| for , and then from |Ψ_*N*_0_,–1/2_⟩⟨Ψ_*N*_0_,–1/2_| to |Ψ_*N*_0_,–3/2_⟩⟨Ψ_*N*_0_,–3/2_|, for .

In contrast, in Case c, the ensemble energy in the presence of the magnetic field becomes  = , being independent of *x*. Therefore, the nonuniqueness of the ground state cannot be removed.

In Case e, the ensemble energy becomes *Ẽ*_ens_(*N*_tot_, *M*_tot_) = (1 − α)*E*(*N*_0_) + α*E*(*N*_0_ + 1) + μ_B_*B*_0_*M*_tot_*F*_*N*_0_,1_ + μ_B_*B*_0_(*F*_*N*_0_,1/2_ − *F*_*N*_0_+1,1_)*y*, and can be minimized with respect to *y*. However, as opposed to Case d, here, a minimal/maximal value of *y* can correspond to a range of possible *x* values; the ground state is not fully determined. This is not surprising, as Case e has Case c as a particular limit for α = 1 (see the SI for details).

Finally, we note that applying a strong magnetic field could further remove the ambiguity in the ground state. However, this scenario may change which pure states contribute to the ensemble ground state, and therefore, it is outside the scope of this Letter.

To conclude, in this Letter we rigorously described the exact ground state of a finite many-electron system, with *N*_tot_ electrons and with spin *M*_tot_, fully employing the ensemble approach. Our results hold for any value of *N*_tot_, any values of the equilibrium spin, *S*_min_(*N*), and for *M*_tot_ confined by eq 4. Description outside Region 4 is a subject for future work.

First, we found that the ground state is an ensemble, which is composed of 2(*S*_0_ + *S*_1_ + 1) pure states, as indicated in eq 5. These states are not necessarily the four integer neighboring points to (*N*_tot_, *M*_tot_) on the *N*_*↑*_ – *N*_*↓*_ grid. The graph of the total energy, *E*_ens_(*N*_tot_, *M*_tot_) consists therefore not only of triangles, but also of trapezoids, when *S*_min_ ⩾ 1. The total energy, the total density, and any quantity *A*, whose pure-state expectation values  do not depend on *M*, are piecewise-linear in *N*_tot_ (i.e., in α) and independent of *M*_tot_.

Second, the highest-occupied KS *↑*- and *↓*-orbital energies are found to be equal strictly within Region 4, and the KS potentials experience a spatially uniform “jump” at the boundary of this region (eq 11), directly related to the spin-migration derivative discontinuity, Δ_sm_^σ^ (eq 11), introduced here for the first time.

Third, we discovered that generally speaking the ground state Γ̂ is not uniquely defined. Unlike the total energy and the total density, which are determined uniquely, the spin-densities are not (eqs 16 and 17). Two ways of removing this nonuniqueness are suggested: adding constraints, e.g., on higher moments of , and minimizing Δ*S*_*z*_. Applying a weak magnetic field does *not* always fully remove the nonuniqueness.

Our findings generalize the piecewise-linearity and the flat-plane conditions and provide new exact properties for many-electron systems. These are expected to be useful in the development of advanced approximations in density functional theory and in other many-electron methods.

## Supporting Information Available

Technical details regarding four subjects: (i) we show that certain pure-state expectation values, including the density  do not depend on *M*; (ii) we discuss the uniqueness of Γ̂ and *Q*_ens_(**r**), given the values of certain moments of ; (iii) we prove that Γ̂ is uniquely determined by the added constraint of minimizing Δ*S*_*z*_; (iv) we elaborate on Cases c, d, and e, regarding the spin-densities, magnetic fields, and the moments of  (PDF)
