# Supplementary material for: Ensemble Ground State of a Many-Electron System with Fractional Electron Number and Spin: Piecewise-Linearity and Flat-Plane Condition Generalized
Source: J Phys Chem Lett. 2024 Feb 22;15(9):2337–43. doi: 10.1021/acs.jpclett.3c03509 (PMC10926161; doi:10.1021/acs.jpclett.3c03509)
Supplement: Supplementary file 1 — jz3c03509_si_001.pdf [file jz3c03509_si_001.pdf]

Supporting Information for:
Ensemble Ground-State of a Many-Electron System With Fractional Electron Number and Spin: Piecewise-Linearity and Flat Plane Condition Generalized

Yuli Goshen[1] and Eli Kraisler[1]

[1] *Fritz Haber Research Center for Molecular Dynamics and Institute of Chemistry, The Hebrew University of Jerusalem, 9091401 Jerusalem, Israel*

(Dated: February 4, 2024)

This document provides further technical details on four subjects. In Sec. I we show that certain pure-state expectation values, including the density, $\langle\Psi_{N,S,M}|\hat{n}(\mathbf{r})|\Psi_{N,S,M}\rangle$, do not depend on M. In Sec. II we show how the ensemble ground state $\hat{\Gamma}$ and/or the spin distribution $Q_{\rm ens}(\mathbf{r})$ can be uniquely determined by setting the higher moments of the operator $\hat{S}_z$. In Sec. III we prove that enforcing the requirement that the deviation ΔS_z is minimal always fully determines the ground state. In Sec. IV we provide a detailed analysis of Cases (c), (d) and (e), which were introduced in the main text, in absence and in presence of a weak, inhomogeneous magnetic field.

In the following, reference to an equation from the main text starts with the letters MT (e.g., Eq. (MT:4)), whereas references to equations within this document are by a single number.

I. DEPENDENCE OF PURE-STATE QUANTITIES ON M

In this section we clarify which pure-state quantities are independent of the value of M, as long as $M \in [-S_{\rm min}, S_{\rm min}]$, and which are not. Consider a quantity described by an operator $\hat{P}$ and compare $P(M) = \langle\Psi_{N,M}|\hat{P}|\Psi_{N,M}\rangle$ with, say, $P(M-1)$. Recall that $\hat{S}_\pm|\Psi_{N,M\mp1}\rangle = \text{const.}|\Psi_{N,M}\rangle$, where $\hat{S}_\pm = \hat{S}_\mp^\dagger = \hat{S}_x \pm i\hat{S}_y$ are the ladder operators, which raise/lower M by 1. Furthermore, note that $|\Psi_{N,M}\rangle$ is an eigenstate of the operator $\hat{S}_-\hat{S}_+$. Then, $P(M-1) = \langle\Psi_{N,M-1}|\hat{P}|\Psi_{N,M-1}\rangle = \frac{\langle\Psi_{N,M}|\hat{S}_+\hat{P}\hat{S}_-|\Psi_{N,M}\rangle}{\langle\Psi_{N,M}|\hat{S}_+\hat{S}_-|\Psi_{N,M}\rangle}$. It then directly follows that for any operator $\hat{P}$ *that commutes with* $\hat{S}_+$, $P(M-1) = P(M)$, i.e., the value of P is M-independent. Such is the case for the Hamiltonian $\hat{H}$, in absence of a magnetic field, for the electron density operator $\hat{n}(\mathbf{r})$, for the reduced density matrix, $\hat{\rho}_1(\mathbf{r},\mathbf{r}')$, and for any operator that depends only on spatial, and not on spin-coordinates. However, this is not the case for the Hamiltonian in presence of a magnetic field, for the spin-density operator $\hat{n}_\sigma(\mathbf{r})$ or for the spin-dependent reduced density matrix $\hat{\gamma}_1(\mathbf{r}\sigma,\mathbf{r}'\sigma')$.

II. HIGHER MOMENTS OF $\hat{S}_z$

In this section we discuss the possibility to determine the ground state $\hat{\Gamma}$ uniquely by choosing the values of moments of $\hat{S}_z$ (i.e. $\langle\hat{S}_z\rangle, \langle\hat{S}_z^2\rangle, \langle\hat{S}_z^3\rangle$, etc.), in addition to Eqs. (MT:1)–(MT:3), and the possibility to determine the spin distribution $Q_{\rm ens}(\mathbf{r}) = n^\uparrow_{\rm ens}(\mathbf{r}) - n^\downarrow_{\rm ens}(\mathbf{r})$, by choosing the values of *odd* moments of $\hat{S}_z$. Recall that the first moment is $M_{\rm tot} = \langle\hat{S}_z\rangle$.

First, consider $N_{\rm tot} \in \mathbb{N}$ (as relevant in Cases (c) and (d) in the main text). There are $2S_0+1$ ensemble coefficients, and the moments of $\hat{S}_z$ are given by

$$\begin{pmatrix} 1 \\ M_{\rm tot} \\ \langle\hat{S}_z^2\rangle \\ \vdots \\ \langle\hat{S}_z^{2S_0}\rangle \end{pmatrix} = \begin{pmatrix} 1 & \cdots & 1 \\ -S_0 & \cdots & S_0 \\ (-S_0)^2 & \cdots & S_0^2 \\ \vdots & & \vdots \\ (-S_0)^{2S_0} & \cdots & S_0^{2S_0} \end{pmatrix} \begin{pmatrix} \gamma_{N_0,-S_0} \\ \gamma_{N_0,-S_0+1} \\ \vdots \\ \vdots \\ \gamma_{N_0,S_0} \end{pmatrix}. \quad (1)$$

The $(2S_0+1)\times(2S_0+1)$ matrix of Eq. (1) is a Vandermonde matrix [1], where no two columns are identical, hence it is invertible. Therefore, the moments of $\hat{S}_z$ up to $\hat{S}_z^{2S_0}$ determine the ensemble coefficients, and thus $\hat{\Gamma}$.

Next we show that $Q_{\rm ens}(\mathbf{r})$ is determined by the *odd* moments, $\langle\hat{S}_z^k\rangle$, where $1 \leqslant k \leqslant 2S_0$. Importantly, the density $n_{\rm ens}(\mathbf{r}) = n^\uparrow_{\rm ens}(\mathbf{r}) + n^\downarrow_{\rm ens}(\mathbf{r})$ is unique, depending only on $N_{\rm tot}$. Thus, if $Q_{\rm ens}(\mathbf{r})$ is determined uniquely (while $\hat{\Gamma}$ may remain undetermined), the spin-densities $n^\sigma_{\rm ens}(\mathbf{r})$ are determined from $Q_{\rm ens}(\mathbf{r})$ uniquely, as well.

If for instance $N_{\rm tot}$ is an odd integer, then $2S_0$ is an odd integer, and the odd moments of $\hat{S}_z$ up to $\langle \hat{S}_z^{2S_0}\rangle$ can be expressed as

$$\begin{pmatrix} M_{\rm tot} \\ \langle \hat{S}_z^3\rangle \\ \langle \hat{S}_z^5\rangle \\ \vdots \\ \langle \hat{S}_z^{2S_0}\rangle \end{pmatrix} = \begin{pmatrix} \frac{1}{2} & \cdots & S_0 \\ \left(\frac{1}{2}\right)^3 & \cdots & S_0^3 \\ \left(\frac{1}{2}\right)^5 & \cdots & S_0^5 \\ \vdots & & \vdots \\ \left(-\frac{1}{2}\right)^{2S_0} & \cdots & S_0^{2S_0} \end{pmatrix} \begin{pmatrix} \gamma_{N_0,1/2} - \gamma_{N_0,-1/2} \\ \gamma_{N_0,3/2} - \gamma_{N_0,-3/2} \\ \gamma_{N_0,5/2} - \gamma_{N_0,-5/2} \\ \vdots \\ \gamma_{N_0,S_0} - \gamma_{N_0,-S_0} \end{pmatrix}. \tag{2}$$

The matrix in Eq. (2) is similar to that of Eq. (1), however the powers in each row are not consecutive integers, hence it is not a Vandermonde matrix but a generalized Vandermonde matrix [1]. This matrix is also invertible, meaning that the odd moments $\langle \hat{S}_z\rangle, \ldots, \langle \hat{S}_z^{2S_0}\rangle$ determine the differences $(\gamma_{N_0,1/2} - \gamma_{N_0,-1/2})$, ..., $(\gamma_{N_0,S_0} - \gamma_{N_0,-S_0})$. Since $Q_{N,M}(\mathbf{r}) = -Q_{N,-M}(\mathbf{r})$, the ensemble spin distribution can be expressed as $Q_{\rm ens}(\mathbf{r}) = \sum_{M=\frac{1}{2}}^{S_0}(\gamma_{N_0,M} - \gamma_{N_0,-M})Q_{N,M}(\mathbf{r})$, meaning it is also determined by the odd moments $\langle \hat{S}_z\rangle, \ldots, \langle \hat{S}_z^{2S_0}\rangle$. One can show in a similar manner that if $N_{\rm tot}$ is an even integer then $Q_{\rm ens}(\mathbf{r})$ is determined by $\langle \hat{S}_z\rangle, \langle \hat{S}_z^3\rangle, \ldots, \langle \hat{S}_z^{2S_0-1}\rangle$.

Next we consider the case of $N_{\rm tot} \notin \mathbb{N}$, where there are now $2(S_0 + S_1 + 1)$ ensemble coefficients. We express the moments of $\hat{S}_z$, as well as α, by

$$\begin{pmatrix} 1 \\ \alpha \\ M_{\rm tot} \\ \langle \hat{S}_z^2\rangle \\ \vdots \\ \langle \hat{S}_z^{2S_0+2S_1}\rangle \end{pmatrix} = \begin{pmatrix} 1 & \cdots & 1 & 1 & \cdots & 1 \\ 0 & \cdots & 0 & 1 & \cdots & 1 \\ -S_0 & \cdots & S_0 & -S_1 & \cdots & S_1 \\ (-S_0)^2 & \cdots & S_0^2 & (-S_1)^2 & \cdots & S_1^2 \\ \vdots & & \vdots & \vdots & & \vdots \\ (-S_0)^{2S_0+2S_1} & \cdots & S_0^{2S_0+2S_1} & -S_1^{2S_0+2S_1} & \cdots & S_1^{2S_0+2S_1} \end{pmatrix} \begin{pmatrix} \gamma_{N_0,-S_0} \\ \vdots \\ \gamma_{N_0,S_0} \\ \gamma_{N_0+1,-S_1} \\ \vdots \\ \gamma_{N_0+1,S_1} \end{pmatrix}. \tag{3}$$

If this matrix is invertible, then indeed the ground state is determined by the values of the moments of $\hat{S}_z$, up to $\langle \hat{S}_z^{2S_0+2S_1}\rangle$, together with α. We have explicitly verified that this is the case for $S_0, S_1 \leqslant 8$, using the `NumPy` [2] library in `Python`. This covers essentially all the chemically relevant scenarios. However, if this matrix is not invertible, then it is sufficient to choose the value of $\langle \hat{S}_z^{2S_0+2S_1+1}\rangle$ as an additional constraint (consistently with all the other constraints). Then,

$$\begin{pmatrix} 1 \\ M_{\rm tot} \\ \langle \hat{S}_z^2\rangle \\ \vdots \\ \langle \hat{S}_z^{2S_0+2S_1+1}\rangle \end{pmatrix} = \begin{pmatrix} 1 & \cdots & 1 & 1 & \cdots & 1 \\ -S_0 & \cdots & S_0 & -S_1 & \cdots & S_1 \\ (-S_0)^2 & \cdots & S_0^2 & (-S_1)^2 & \cdots & S_1^2 \\ \vdots & & \vdots & \vdots & & \vdots \\ (-S_0)^{2S_0+2S_1+1} & \cdots & S_0^{2S_0+2S_1+1} & (-S_1)^{2S_0+2S_1+1} & \cdots & S_1^{2S_0+2S_1+1} \end{pmatrix} \begin{pmatrix} \gamma_{N_0,-S_0} \\ \vdots \\ \gamma_{N_0,S_0} \\ \gamma_{N_0+1,-S_1} \\ \vdots \\ \gamma_{N_0+1,S_1} \end{pmatrix}. \tag{4}$$

The matrix of Eq. (4) is another Vandermonde matrix, hence it is invertible.

To determine $Q_{\rm ens}(\mathbf{r})$, now with $N_{\rm tot} \notin \mathbb{N}$, assume that N_0 is odd. Then, analogously to Eq. (2),

$$\begin{pmatrix} M_{\rm tot} \\ \langle \hat{S}_z^3\rangle \\ \langle \hat{S}_z^5\rangle \\ \vdots \\ \langle \hat{S}_z^{2S_0+2S_1}\rangle \end{pmatrix} = \begin{pmatrix} \frac{1}{2} & \cdots & S_0 & 1 & \cdots & S_1 \\ \left(\frac{1}{2}\right)^3 & \cdots & S_0^3 & 1^3 & \cdots & S_1^3 \\ \left(\frac{1}{2}\right)^5 & \cdots & S_0^5 & 1^5 & \cdots & S_1^5 \\ \vdots & & \vdots & \vdots & & \vdots \\ \left(\frac{1}{2}\right)^{2S_0+2S_1} & \cdots & S_0^{2S_0+2S_1} & 1^{2S_0+2S_1} & \cdots & S_1^{2S_0+2S_1} \end{pmatrix} \begin{pmatrix} \gamma_{N_0,1/2} - \gamma_{N_0,-1/2} \\ \vdots \\ \gamma_{N_0,S_0} - \gamma_{N_0,-S_0} \\ \gamma_{N_0+1,1} - \gamma_{N_0+1,-1} \\ \vdots \\ \gamma_{N_0+1,S_1} - \gamma_{N_0+1,-S_1} \end{pmatrix}. \tag{5}$$

The matrix in Eq. (5) is another generalized Vandermonde matrix, meaning it is invertible. Since $Q_{\rm ens}(\mathbf{r}) = \sum_{N=N_0}^{N_0+1}\sum_{M>0}(\gamma_{N,M} - \gamma_{N,-M})Q_{N,M}(\mathbf{r})$, we see that $Q_{\rm ens}(\mathbf{r})$ is fully determined by the odd moments of $\hat{S}_z$, up to $\langle \hat{S}_z^{2S_0+2S_1}\rangle$. This can be shown for the case of N_0 being even, in the same way.

III. MINIMIZING ΔS_z

In this section we prove that $\hat{\Gamma}$ is determined uniquely by adding the constraint of minimizing ΔS_z, or equivalently, minimizing $\langle \hat{S}_z^2\rangle$, as $\langle \hat{S}_z\rangle = M_{\rm tot}$ is given by

Eq. (MT:2). Consider an ensemble $\hat{\Gamma}$ (Eq. (MT:5)), which minimizes $\langle \hat{S}_z^2 \rangle$. Since the ensemble coefficients are all between 0 and 1, we may write them as $\gamma_{N,M} = \sin^2(\theta_{N,M})$. Using Lagrange multipliers, with Eqs. (MT:1)–(MT:3) as constraints, we find

$$\frac{\partial}{\partial \theta_{N,M}} \left(\sum_{N'=N_0}^{N_0+1} \sum_{M'=-S_{\min}(N')}^{S_{\min}(N')} \gamma_{N',M'} M'^2 \right) = \kappa_1 \frac{\partial}{\partial \theta_{N,M}} \left(\sum_{N'=N_0}^{N_0+1} \sum_{M'=-S_{\min}(N')}^{S_{\min}(N')} \gamma_{N',M'} \right) + \\ + \kappa_2 \frac{\partial}{\partial \theta_{N,M}} \left(\sum_{N'=N_0}^{N_0+1} \sum_{M'=-S_{\min}(N')}^{S_{\min}(N')} \gamma_{N',M'} N' \right) + \kappa_3 \frac{\partial}{\partial \theta_{N,M}} \left(\sum_{N'=N_0}^{N_0+1} \sum_{M'=-S_{\min}(N')}^{S_{\min}(N')} \gamma_{N',M'} M' \right) \quad (6)$$

where $\kappa_1, \kappa_2, \kappa_3$ are some constants. Taking the derivatives, we have $2 \sin\theta_{N,M} \cos\theta_{N,M} (M^2 - \kappa_1 + \kappa_2 N + \kappa_3 M) = 0$. Hence, for each N, M such that $\gamma_{N,M} \neq 0, 1$, we have

$$M^2 = \kappa_1 + \kappa_2 N + \kappa_3 M. \quad (7)$$

Since Eq. (7) is quadratic in M, there are at most two solutions for each value of N. This means there are at most four nonzero coefficients - $\gamma_{N_0,a}, \gamma_{N_0,b}, \gamma_{N_0+1,c}, \gamma_{N_0+1,d}$. Here $M = a, b$ are the solutions to Eq. (7) for $N = N_0$, and $M = c, d$ are the solutions for $N = N_0 + 1$. If Eq. (7) has only one solution for $N = N_0$ or $N = N_0 + 1$, then $a = b$ or $c = d$, respectively. In addition, from Eq. (7) we have

$$a + b = c + d = \kappa_3. \quad (8)$$

(i) Consider $N_{\text{tot}} \in \mathbb{N}$. Due to Eqs. (MT:1), (MT:3), we have $\gamma_{N_0+1,c} = \gamma_{N_0+1,d} = 0$, and only $\gamma_{N_0,a}$ and $\gamma_{N_0,b}$ may be nonzero. Hence, $\hat{\Gamma} = \gamma_{N_0,a} |\Psi_{N_0,a}\rangle\langle\Psi_{N_0,a}| + \gamma_{N_0,b} |\Psi_{N_0,b}\rangle\langle\Psi_{N_0,b}|$. If M_{tot} is an integer (for an even N_{tot}) or a half-integer (for an odd N_{tot}), then ΔS_z is minimized only by $|\Psi_{N_{\text{tot}},M_{\text{tot}}}\rangle\langle\Psi_{N_{\text{tot}},M_{\text{tot}}}|$, as $\Delta S_z = 0$ in this case. Otherwise, $\gamma_{N_0,a}, \gamma_{N_0,b}$ are both nonzero, and $a \neq b$. From Eqs. (MT:2) and (MT:3), we have $\gamma_a = \frac{1}{a-b}(M_{\text{tot}} - b)$ and $\gamma_b = \frac{1}{a-b}(a - M_{\text{tot}})$. Therefore

$$\langle \hat{S}_z^2 \rangle = \gamma_{N_0,a} a^2 + \gamma_{N_0,b} b^2 = M_{\text{tot}} (a + b) - ab. \quad (9)$$

Without loss of generality, we assume that $b < a$. Furthermore, since $M_{\text{tot}} = \gamma_{N_0,a} a + \gamma_{N_0,b} b$, and all $\gamma_{N,M}$'s are within $[0, 1]$, we realize that $b < M_{\text{tot}} < a$. By rewriting Eq. (9) as $\langle \hat{S}_z^2 \rangle = (M_{\text{tot}} - b)\, a + M_{\text{tot}} b$, we see that $\langle \hat{S}_z^2 \rangle$ is minimized by minimizing a. Similarly, by writing $\langle \hat{S}_z^2 \rangle = (M_{\text{tot}} - a)\, b + M_{\text{tot}} a$, we see that $\langle \hat{S}_z^2 \rangle$ is minimized by maximizing b. Therefore a is the smallest integer or half-integer (in accordance with N_0) greater than M_{tot}, and b is the largest integer/half-integer smaller than M_{tot}. In particular, $a = b + 1$. Therefore, the ground state minimizing $\langle \hat{S}_z^2 \rangle$ is unique in this case.

(ii) Consider the case $N_{\text{tot}} \notin \mathbb{N}$. We define $\hat{\Gamma}_0 = \frac{1}{1-\alpha}(\gamma_{N_0,a} |\Psi_{N_0,a}\rangle\langle\Psi_{N_0,a}| + \gamma_{N_0,b} |\Psi_{N_0,b}\rangle\langle\Psi_{N_0,b}|)$, $\hat{\Gamma}_1 = \frac{1}{\alpha}(\gamma_{N_0+1,c} |\Psi_{N_0+1,c}\rangle\langle\Psi_{N_0+1,c}| + \gamma_{N_0+1,d} |\Psi_{N_0+1,d}\rangle\langle\Psi_{N_0+1,d}|)$, $M_0 = \text{Tr}\left\{\hat{\Gamma}_0 \hat{S}_z\right\}$ and $M_1 = \text{Tr}\left\{\hat{\Gamma}_1 \hat{S}_z\right\}$. Then $\hat{\Gamma} = (1 - \alpha)\hat{\Gamma}_0 + \alpha\hat{\Gamma}_1$, and $\text{Tr}\left\{\hat{\Gamma}\hat{S}_z^2\right\} = (1 - \alpha)\, \text{Tr}\left\{\hat{\Gamma}_0 \hat{S}_z^2\right\} + \alpha\, \text{Tr}\left\{\hat{\Gamma}_1 \hat{S}_z^2\right\}$. Therefore a, b must minimize $\text{Tr}\left\{\hat{\Gamma}_0 \hat{S}_z^2\right\}$ and c, d must minimize $\text{Tr}\left\{\hat{\Gamma}_1 \hat{S}_z^2\right\}$, under the constraints $\text{Tr}\left\{\hat{\Gamma}_0 \hat{S}_z\right\} = M_0$ and $\text{Tr}\left\{\hat{\Gamma}_1 \hat{S}_z\right\} = M_1$.

Assume, aiming for contradiction, that $a \neq b$ and $c \neq d$. Without loss of generality assume that $a > b$ and $c > d$. Therefore, applying the result of (i) to $\hat{\Gamma}_0$ and $\hat{\Gamma}_1$, we find that $a = b + 1$ and $c = d + 1$, hence $a + b - c - d = 2(b - d)$. Since b is the spin of an N_0-electron system, and d is the spin of an $N_0 + 1$-electron system, $b - d$ is a half-integer and therefore $b - d \neq 0$, meaning $a + b - c - d \neq 0$. This is a contradiction to Eq. (8). Therefore, by minimizing ΔS_z, we are left with at most three nonzero $\gamma_{N,M}$'s, which can then be derived from Eqs. (MT:1)–(MT:3), uniquely. Particularly, in case $\gamma_{N_0+1,d} = 0$ and is ruled out, Eqs. (MT:1)–(MT:3) read:

$$\begin{pmatrix} 1 \\ \alpha \\ M_{\text{tot}} \end{pmatrix} = \begin{pmatrix} 1 & 1 & 1 \\ 0 & 0 & 1 \\ a & b & c \end{pmatrix} \begin{pmatrix} \gamma_{N_0,a} \\ \gamma_{N_0,b} \\ \gamma_{N_0+1,c} \end{pmatrix}. \quad (10)$$

The determinant of the matrix above equals $a - b = 1$. Therefore, the matrix is invertible. If instead both $\gamma_{N_0+1,c}$ and $\gamma_{N_0+1,d}$ are present, but $\gamma_{N_0,b}$ vanishes, the corresponding matrix is invertible, as well.

Finally, we show that a, b and c are uniquely determined. Assume by contradiction that there exists another ensemble state $\hat{\Gamma}'$, which also minimizes $\langle \hat{S}_z^2 \rangle$ under the constraints (MT:1)–(MT:3). Thus, there is a different triad a', b' and c', which corresponds to a minimum. But then we may refer to $\hat{\Gamma}'' = \frac{1}{2}(\hat{\Gamma} + \hat{\Gamma}')$: it also minimizes $\langle \hat{S}_z^2 \rangle$ under the same constraints, but consists of *more* than three pure states, which is a contradiction. In case the nonzero ensemble coefficients are, e.g., $\gamma_{N_0,a}$, $\gamma_{N_0+1,c}$ and $\gamma_{N_0+1,d}$, the same logic applies.

IV. ANALYSIS OF CASES (C), (D) AND (E)

In this section we present in detail Cases (c), (d) and (e), which were introduced in the main text. We start by considering spin migration at integer electron number, namely, the situation when $N_{\rm tot} = N_0 = $ const., i.e. $\alpha = 0$ and $M_{\rm tot} \in [-S_0, S_0]$. As mentioned in the main text, from Eq. (MT:9) we realize that $\sum_{M=-S_1}^{S_1} \gamma_{N_0+1,M} = 0$, and since $\gamma_{N,M} \in [0,1]$, it follows that $\gamma_{N_0+1,M} = 0$ for all M. As we are left with $\gamma_{N_0,M}$ only, for brevity, we may omit in what follows the index N_0. Yet, when it serves clarity we add it back. From Eq. (MT:9) we then get $\sum_{M=-S_0}^{S_0} \gamma_M = 1$ and $\sum_{M=-S_0}^{S_0} M\gamma_M = M_{\rm tot}$ – two equations to determine $(2S_0+1)$ coefficients. We therefore expect that for $S_0 \geqslant 1$ the coefficients $\gamma_{N,M}$, and therefore the ensemble state $\hat{\Gamma}$, would not be determined unambiguously.

We now refer to specific scenarios of spin migration, for selected values of S_0. Trivially, for $S_0 = 0$, there is no spin migration at all, $\gamma_0 = 1$ and $M_{\rm tot}$ must equal 0.

For $S_0 = \frac{1}{2}$ (e.g., spin migration for the H or for the Li atom), which is a particular scenario within Case (a), we find that

$$\begin{aligned} \gamma_{1/2} =& \frac{1}{2} + M_{\rm tot} \\ \gamma_{-1/2} =& \frac{1}{2} - M_{\rm tot}. \end{aligned} \tag{11}$$

The coefficients are determined unambiguously, and so are all the quantities that describe the system. In particular, $E_{\rm ens}(N_{\rm tot}, M_{\rm tot}) = E(N_0)$, $\langle \hat{S}_z \rangle = M_{\rm tot}$, $\langle \hat{S}_z^2 \rangle = \frac{1}{4}$ – independent of $M_{\rm tot}$. More generally, all the even moments of $\hat{S}_z$, $\langle \hat{S}_z^{2k} \rangle = (\frac{1}{2})^{2k}$ are $M_{\rm tot}$-independent and all the odd moments $\langle \hat{S}_z^{2k+1} \rangle = (\frac{1}{2})^{2k} M_{\rm tot}$ are linear in $M_{\rm tot}$ ($k \in \mathbb{N}$). The deviation in $\hat{S}_z$ is $\Delta S_z := \sqrt{\langle \hat{S}_z^2 \rangle - \langle \hat{S}_z \rangle^2} = \sqrt{\frac{1}{4} - M_{\rm tot}^2}$.

To find the ensemble spin-densities, $n^\sigma_{\rm ens}(\mathbf{r})$, we recall that: (i) for any pure-state, $|\Psi_{N,M}\rangle$, the spin-densities relate as $n^{\downarrow}_{N,M}(\mathbf{r}) = n^{\uparrow}_{N,-M}(\mathbf{r})$; (ii) the total density is M-independent (see Sec. I above). Then,

$$n^\sigma_{\rm ens}(\mathbf{r}) = \frac{1}{2} n_{N_0}(\mathbf{r}) + \frac{1}{2}\delta_\sigma \frac{M_{\rm tot}}{S_0} Q_{N_0,S_0}(\mathbf{r}), \tag{12}$$

where

$$\delta_\sigma = \left\{ \begin{array}{rcl} 1 & : & \sigma = \uparrow \\ -1 & : & \sigma = \downarrow \end{array} \right. , \tag{13}$$

and $Q(\mathbf{r}) := n^\uparrow(\mathbf{r}) - n^\downarrow(\mathbf{r})$ is the spin distribution. From Eq. (12) it directly follows that the total ensemble density $n_{\rm ens}(\mathbf{r}) = n_{N_0}(\mathbf{r})$ is $M_{\rm tot}$-independent and the spin distribution

$$Q_{\rm ens}(\mathbf{r}) = \frac{M_{\rm tot}}{S_0} Q_{N_0,S_0}(\mathbf{r}), \tag{14}$$

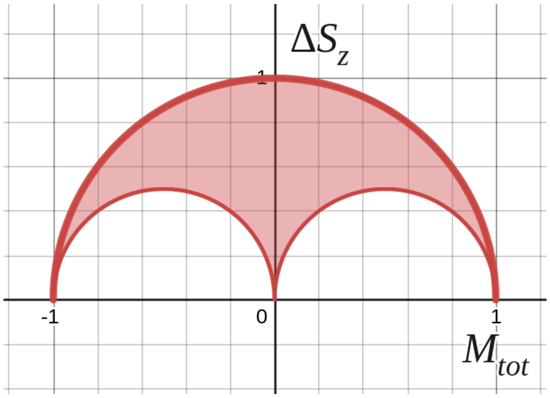

FIG. 1. The deviation ΔS_z versus $M_{\rm tot}$, for $S_0 = 1$. Minimal deviation $(\Delta S_z)_{\rm min}$: solid thin line, maximal deviation $(\Delta S_z)_{\rm max}$: solid thick line.

is linear in $M_{\rm tot}$. For all cases of integer $N_{\rm tot}$, the spin polarization, $\zeta(\mathbf{r}) = \frac{n^\uparrow(\mathbf{r}) - n^\downarrow(\mathbf{r})}{n^\uparrow(\mathbf{r}) + n^\downarrow(\mathbf{r})}$, has an analogous form to that of $Q_{\rm ens}(\mathbf{r})$.

For $S_0 = 1$ (e.g., spin migration for the C atom), which is **Case (c)** of the main text, we obtain three coefficients that are not identically zero. They can be expressed as:

$$\begin{aligned} \gamma_1 =& \frac{1}{2}x + \frac{1}{2}M_{\rm tot} \\ \gamma_0 =& 1 - x \\ \gamma_{-1} =& \frac{1}{2}x - \frac{1}{2}M_{\rm tot} \end{aligned} \tag{15}$$

where x remains undetermined. The value of x is not arbitrary, though: it is confined by the requirement that all $\gamma_{N,M} \in [0,1]$. Applying this requirement to all three coefficients above, we find that $x \in [\,|M_{\rm tot}|, 1]$.

Whereas the total energy, $E_{\rm ens}(N_{\rm tot}, M_{\rm tot}) = E(N_0)$ and all the odd moments of $\hat{S}_z$, $\langle \hat{S}_z^{2k+1} \rangle = M_{\rm tot}$, are determined uniquely, the even moments of $\hat{S}_z$ equal $\langle \hat{S}_z^{2k} \rangle = x$. Therefore, to fully determine the ensemble ground state for this Case, it is possible to add a requirement as to the value of, e.g., $\langle \hat{S}_z^2 \rangle$ (in agreement with the conclusions of Sec. II above).

Alternatively, it is possible to restrict the deviation, ΔS_z, to be minimal. Accounting for the aforementioned range of the variable x, we find that $(\Delta S_z)_{\rm min} = \sqrt{|M_{\rm tot}| - M_{\rm tot}^2}$, as depicted in Fig. 1. The ensemble coefficients in this case equal

$$\begin{aligned} \gamma_1 &= \frac{1}{2}(|M_{\rm tot}| + M_{\rm tot}) = \left\{ \begin{array}{rcl} 0 & : & M_{\rm tot} < 0 \\ M_{\rm tot} & : & M_{\rm tot} \geqslant 0 \end{array} \right. \\ \gamma_0 &= 1 - |M_{\rm tot}| \\ \gamma_{-1} &= \frac{1}{2}(|M_{\rm tot}| - M_{\rm tot}) = \left\{ \begin{array}{rcl} -M_{\rm tot} & : & M_{\rm tot} < 0 \\ 0 & : & M_{\rm tot} \geqslant 0 \end{array} \right. \end{aligned} \tag{16}$$

(see Fig. 2). Note that for every value of $M_{\rm tot}$ we obtain two nonzero ensemble coefficients at most. Therefore, as

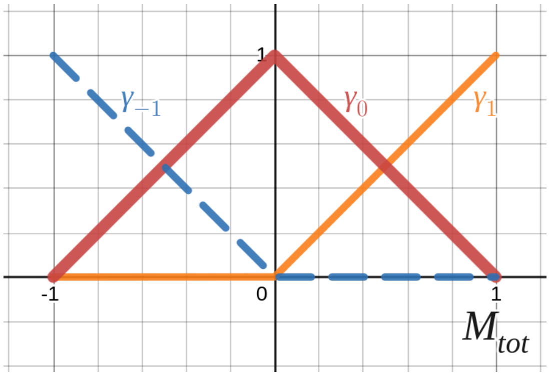

FIG. 2. The ensemble coefficients, γ_{-1} (dashed blue), γ_0 (solid thick red) and γ_1 (solid orange), versus $M_{\rm tot}$, for $S_0 = 1$, for the scenario of $(\Delta S_z)_{\rm min}$.

we increase $M_{\rm tot}$ from -1 to 0, we see a linear transition from the pure state $|\Psi_{N_0,-1}\rangle\langle\Psi_{N_0,-1}|$ to $|\Psi_{N_0,0}\rangle\langle\Psi_{N_0,0}|$; further increase of $M_{\rm tot}$ from 0 to 1 gives a linear transition from $|\Psi_{N_0,0}\rangle\langle\Psi_{N_0,0}|$ to $|\Psi_{N_0,1}\rangle\langle\Psi_{N_0,1}|$. At the points $M_{\rm tot} = -1, 0, 1$, we have pure states (only one coefficient is nonzero) and therefore the deviation ΔS_z vanishes (see Fig. 1).

For completeness, consider the scenario where ΔS_z is taken to be maximal. Then, $x = 1$ and $(\Delta S_z)_{\rm max} = \sqrt{1 - M_{\rm tot}^2}$ (depicted in Fig. 1, as well). For such a case, $\gamma_1 = \frac{1}{2}(1 + M_{\rm tot})$, $\gamma_0 = 0$, $\gamma_{-1} = \frac{1}{2}(1 - M_{\rm tot})$, and we obtain, upon increase of $M_{\rm tot}$, a gradual transition between $|\Psi_{N_0,-1}\rangle\langle\Psi_{N_0,-1}|$ and $|\Psi_{N_0,1}\rangle\langle\Psi_{N_0,1}|$ (excluding $|\Psi_{N_0,0}\rangle\langle\Psi_{N_0,0}|$).

The spin-densities for this Case can be written at first as $n^\sigma_{\rm ens}(\mathbf{r}) = \frac{1}{2}(1 - x)n_{N_0,0}(\mathbf{r}) + \frac{1}{2}x\, n_{N_0,1}(\mathbf{r}) + \frac{1}{2}\delta_\sigma M_{\rm tot} Q_{N_0,1}(\mathbf{r})$, but since the pure-state densities are M-independent, it directly follows that, similarly to the $S_0 = \frac{1}{2}$ case, $n^\sigma_{\rm ens}(\mathbf{r})$ satisfies Eq. (12) and does not depend on x, and $Q_{\rm ens}(\mathbf{r})$ satisfies Eq. (14).

In presence of a magnetic field, the total energy in Case (c) changes linearly with $M_{\rm tot}$, as described in the main text. It does not depend, however, on x, and therefore cannot be minimized with respect to x. Surprisingly, introduction of a magnetic field does not remove the ambiguity in the ground state, for this Case. Therefore, in this respect, the requirement on ΔS_z is stronger.

For $S_0 = \frac{3}{2}$ (e.g., spin migration for the N atom), which is **Case (d)** of the main text, we obtain four coefficients, which are not identically zero. They can be expressed using the variables x and y:

$$\begin{aligned}\gamma_{3/2} =& \frac{1}{2}x + \frac{1}{3}(M_{\rm tot} - y)\\ \gamma_{1/2} =& \frac{1}{2}(1 - x) + y\\ \gamma_{-1/2} =& \frac{1}{2}(1 - x) - y\\ \gamma_{-3/2} =& \frac{1}{2}x - \frac{1}{3}(M_{\rm tot} - y).\end{aligned} \tag{17}$$

Whereas $E_{\rm ens}(N_{\rm tot}, M_{\rm tot}) = E(N_0)$ and $\langle\hat{S}_z\rangle = M_{\rm tot}$ are determined uniquely, other quantities that describe the system are not. The even moments of $\hat{S}_z$ equal $\langle\hat{S}_z^{2k}\rangle = S_0^{2k}x + (S_0 - 1)^{2k}(1 - x)$, and are linear in x and independent of y, whereas the odd moments of $\hat{S}_z$ are $\langle\hat{S}_z^{2k+1}\rangle = S_0^{2k}(M_{\rm tot}-y)+(S_0-1)^{2k}y$ and are linear in y and independent of x. To fully determine the ensemble ground state for this Case, we can add two constraints, e.g., for $\langle\hat{S}_z^2\rangle$ and $\langle\hat{S}_z^3\rangle$.

As opposed to previous cases, here even the spin-densities are not determined unambiguously. They can be expressed as

$$n^\sigma_{\rm ens}(\mathbf{r}) = \frac{1}{2}n_{N_0}(\mathbf{r}) + \frac{1}{2}\delta_\sigma M_{\rm tot}\frac{Q_{N_0,3/2}(\mathbf{r})}{3/2} + + \frac{1}{2}\delta_\sigma y\left(\frac{Q_{N_0,1/2}(\mathbf{r})}{1/2} - \frac{Q_{N_0,3/2}(\mathbf{r})}{3/2}\right), \tag{18}$$

being independent of x and linear in y. The spin distribution $Q_{\rm ens}(\mathbf{r})$ can then be written as

$$Q_{\rm ens}(\mathbf{r}) = (M_{\rm tot} - y)\frac{Q_{N_0,3/2}(\mathbf{r})}{3/2} + y\frac{Q_{N_0,1/2}(\mathbf{r})}{1/2}; \tag{19}$$

it is linear in y, as well. For $y = 0$ it reduces to Eq. (14).

As we already mentioned above, one of the ways to fully determine the ground state is to require that the deviation ΔS_z is minimal. In our Case, $\Delta S_z = \sqrt{\frac{1}{4} - M_{\rm tot}^2 + 2x}$, which means that the minimal value of ΔS_z is obtained for the minimal value of x. As before, the domain of x (and y) is restricted by the requirement that all the ensemble coefficients are within $[0, 1]$. Superimposing all eight conditions that emerge from this requirement, we are left with two inequalities: $|y| \leqslant \frac{1}{2}(1 - x)$ and $|y - M_{\rm tot}| \leqslant \frac{3}{2}x$. Graphically, the domain of x and y (which parametrically depends on $M_{\rm tot}$) is illustrated in Fig. 3.

From Fig. 3 we conclude that the lowest value of x is

$$x_{\rm min}(M_{\rm tot}) = \begin{cases} -M_{\rm tot} - \frac{1}{2} &: M_{\rm tot} \in [-\frac{3}{2}, -\frac{1}{2}] \\ 0 &: M_{\rm tot} \in [-\frac{1}{2}, \frac{1}{2}] \\ M_{\rm tot} - \frac{1}{2} &: M_{\rm tot} \in [\frac{1}{2}, \frac{3}{2}] \end{cases}. \tag{20}$$

Observing the geometry of the $x-y$ domain, we find that for each value of $x_{\rm min}$ there is *one* corresponding value of y:

$$y_0(M_{\rm tot}) := y(x_{\rm min}) = \begin{cases} -\frac{3}{4} - \frac{1}{2}M_{\rm tot} &: M_{\rm tot} \in [-\frac{3}{2}, -\frac{1}{2}] \\ M_{\rm tot} &: M_{\rm tot} \in [-\frac{1}{2}, \frac{1}{2}] \\ \frac{3}{4} - \frac{1}{2}M_{\rm tot} &: M_{\rm tot} \in [\frac{1}{2}, \frac{3}{2}] \end{cases}. \tag{21}$$

Therefore, by minimization of ΔS_z, the ground state is defined completely, in agreement with Sec. III. The dependence of $x_{\rm min}$ and y_0 on $M_{\rm tot}$ is depicted on Figs. 4 and 5, respectively.

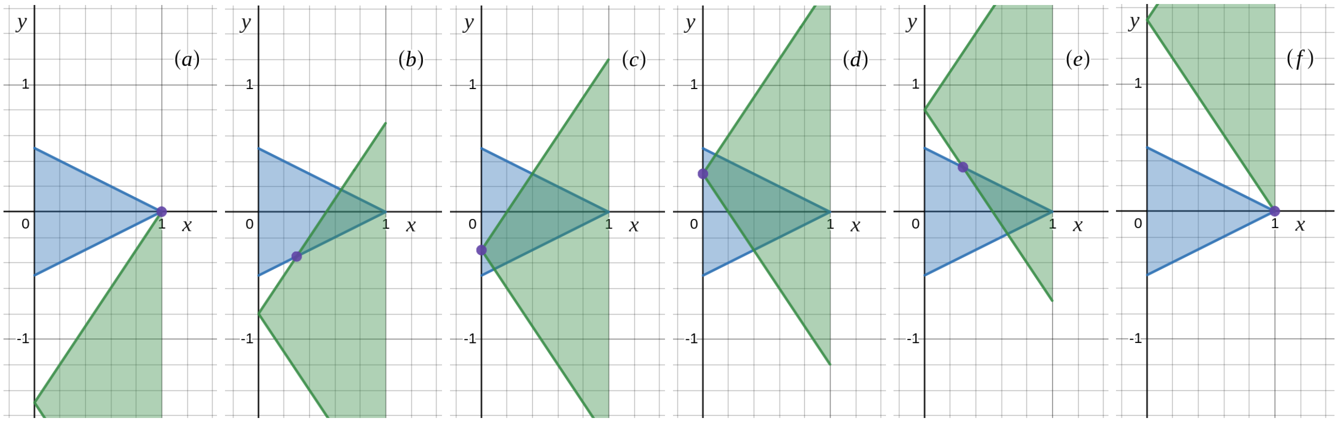

FIG. 3. The allowed domain of x and y, for $S_0 = \frac{3}{2}$, is the intersection of the green and blue areas. In (a), $M_{\rm tot} = -1.5$; in (b), $M_{\rm tot} = -0.8$; in (c), $M_{\rm tot} = -0.3$; in (d), $M_{\rm tot} = 0.3$; in (e), $M_{\rm tot} = 0.8$; in (f), $M_{\rm tot} = 1.5$. The lowest possible value of x is denoted by a purple point. For an interactive plot, see: https://www.desmos.com/calculator/bndzdulimf.

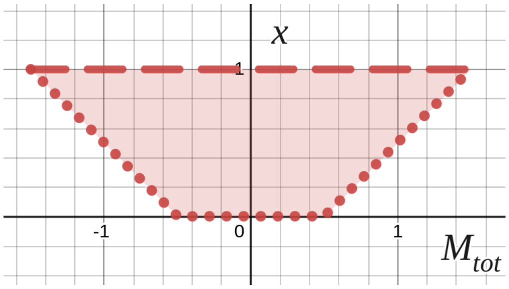

FIG. 4. Dependence of the minimal and the maximal values of x ($x_{\rm min}$, dotted and $x_{\rm max}$, dashed) on $M_{\rm tot}$, for $S_0 = \frac{3}{2}$.

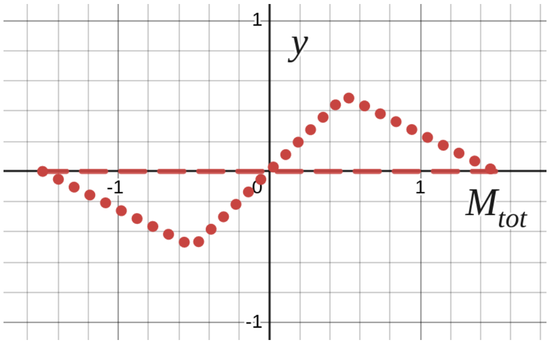

FIG. 5. Dependence of the y-values, which correspond to the minimal and the maximal values of x ($y_0 = y(x_{\rm min})$, dotted and $y_1 = y(x_{\rm max})$, dashed) on $M_{\rm tot}$, for $S_0 = \frac{3}{2}$.

For completeness, let us note that the maximal value of x is $x_{\rm max} = 1$, for any $M_{\rm tot}$, and $y_1 = y(x_{\rm max}) = 0$. Therefore, in this case also maximizing ΔS_z sets both x and y and fully determines the ensemble ground state.

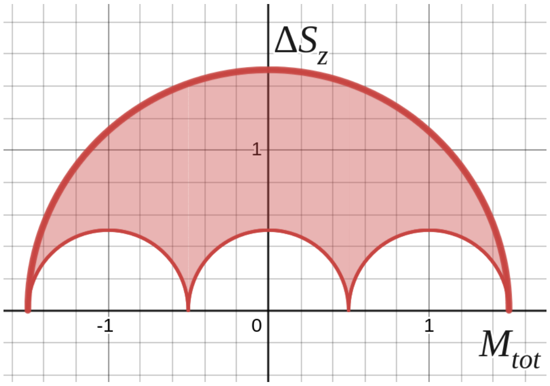

FIG. 6. Deviation ΔS_z versus $M_{\rm tot}$, for $S_0 = \frac{3}{2}$. Minimal deviation, $(\Delta S_z)_{\rm min}$: solid thin line, maximal deviation $(\Delta S_z)_{\rm max}$: solid thick line.

The minimal value of ΔS_z is

$$(\Delta S_z)_{\rm min} = \begin{cases} \sqrt{(M_{\rm tot}+\frac{1}{2})(-M_{\rm tot}-\frac{3}{2})} & : M_{\rm tot} \in [-\frac{3}{2}, -\frac{1}{2}] \\ \sqrt{\frac{1}{4} - M_{\rm tot}^2} & : M_{\rm tot} \in [-\frac{1}{2}, \frac{1}{2}] \\ \sqrt{(M_{\rm tot}-\frac{1}{2})(-M_{\rm tot}+\frac{3}{2})} & : M_{\rm tot} \in [\frac{1}{2}, \frac{3}{2}] \end{cases} \quad (22)$$

and the maximal value is $(\Delta S_z)_{\rm max} = \sqrt{\frac{9}{4} - M_{\rm tot}^2}$; see Fig. 6.

For the scenario of minimal ΔS_z, the ensemble coeffi-

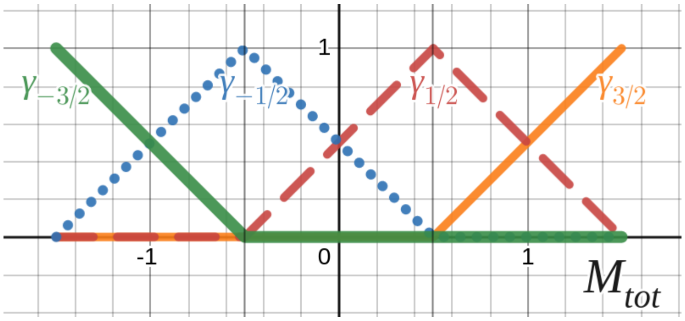

FIG. 7. Ensemble coefficients, $\gamma_{-3/2}$ (thick solid green), $\gamma_{-1/2}$ (dotted blue), $\gamma_{1/2}$ (dashed red) and $\gamma_{3/2}$ (solid orange), versus $M_{\rm tot}$, for $S_0 = \frac{3}{2}$, for the scenario of $(\Delta S_z)_{\rm min}$.

cients equal

$$\gamma_{3/2} = \begin{cases} 0 & : \ M_{\rm tot} \in [-\frac{3}{2}, -\frac{1}{2}] \\ 0 & : \ M_{\rm tot} \in [-\frac{1}{2}, \frac{1}{2}] \\ M_{\rm tot} - \frac{1}{2} & : \ M_{\rm tot} \in [\frac{1}{2}, \frac{3}{2}] \end{cases}$$
$$\gamma_{1/2} = \begin{cases} 0 & : \ M_{\rm tot} \in [-\frac{3}{2}, -\frac{1}{2}] \\ \frac{1}{2} + M_{\rm tot} & : \ M_{\rm tot} \in [-\frac{1}{2}, \frac{1}{2}] \\ \frac{3}{2} - M_{\rm tot} & : \ M_{\rm tot} \in [\frac{1}{2}, \frac{3}{2}] \end{cases} \quad (23)$$
$$\gamma_{-1/2} = \begin{cases} \frac{3}{2} + M_{\rm tot} & : \ M_{\rm tot} \in [-\frac{3}{2}, -\frac{1}{2}] \\ \frac{1}{2} - M_{\rm tot} & : \ M_{\rm tot} \in [-\frac{1}{2}, \frac{1}{2}] \\ 0 & : \ M_{\rm tot} \in [\frac{1}{2}, \frac{3}{2}] \end{cases}$$
$$\gamma_{-3/2} = \begin{cases} -\frac{1}{2} - M_{\rm tot} & : \ M_{\rm tot} \in [-\frac{3}{2}, -\frac{1}{2}] \\ 0 & : \ M_{\rm tot} \in [-\frac{1}{2}, \frac{1}{2}] \\ 0 & : \ M_{\rm tot} \in [\frac{1}{2}, \frac{3}{2}] \end{cases}$$

and are depicted in Fig. 7. Similarly to Case (c), for every value of $M_{\rm tot}$ we obtain two nonzero ensemble coefficients, at most. Therefore, as we increase $M_{\rm tot}$, we find a linear transition from $|\Psi_{N_0,-3/2}\rangle\langle\Psi_{N_0,-3/2}|$ to $|\Psi_{N_0,-1/2}\rangle\langle\Psi_{N_0,-1/2}|$ and then from $|\Psi_{N_0,-1/2}\rangle\langle\Psi_{N_0,-1/2}|$ to $|\Psi_{N_0,1/2}\rangle\langle\Psi_{N_0,1/2}|$ and finally from $|\Psi_{N_0,1/2}\rangle\langle\Psi_{N_0,1/2}|$ to $|\Psi_{N_0,3/2}\rangle\langle\Psi_{N_0,3/2}|$.

The behavior for the moments of $\hat{S}_z$ is piecewise-linear in $M_{\rm tot}$. The even moments are

$$\langle \hat{S}_z^{2k} \rangle = \begin{cases} (S_0-1)^{2k}(\frac{3}{2}+M_{\rm tot}) - S_0^{2k}(\frac{1}{2}+M_{\rm tot}) & : \ M_{\rm tot} \in [-\frac{3}{2}, -\frac{1}{2}] \\ (S_0-1)^{2k} & : \ M_{\rm tot} \in [-\frac{1}{2}, \frac{1}{2}] \\ (S_0-1)^{2k}(\frac{3}{2}-M_{\rm tot}) + S_0^{2k}(M_{\rm tot} - \frac{1}{2}) & : \ M_{\rm tot} \in [\frac{1}{2}, \frac{3}{2}] \end{cases} \quad (24)$$

and have the muffin-tin shape, whereas the odd moments are

$$\langle \hat{S}_z^{2k+1} \rangle = \begin{cases} -(S_0-1)^{2k+1} + \left[S_0^{2k+1} - (S_0-1)^{2k+1}\right](M_{\rm tot}+\frac{1}{2}) & : M_{\rm tot} \in [-\frac{3}{2}, -\frac{1}{2}] \\ (S_0 - 1)^{2k} M_{\rm tot} & : M_{\rm tot} \in [-\frac{1}{2}, \frac{1}{2}] \\ (S_0-1)^{2k+1} + \left[S_0^{2k+1} - (S_0-1)^{2k+1}\right](M_{\rm tot}-\frac{1}{2}) & : M_{\rm tot} \in [\frac{1}{2}, \frac{3}{2}] \end{cases} . \quad (25)$$

Finally, the spin distribution for the case of minimal ΔS_z equals

$$Q_{\rm ens}(\mathbf{r}) = \begin{cases} -Q_{1/2}(\mathbf{r}) + (M_{\rm tot} + \frac{1}{2})(Q_{3/2}(\mathbf{r}) - Q_{1/2}(\mathbf{r})) & : M_{\rm tot} \in [-\frac{3}{2}, -\frac{1}{2}] \\ \frac{M_{\rm tot}}{1/2} Q_{1/2}(\mathbf{r}) & : M_{\rm tot} \in [-\frac{1}{2}, \frac{1}{2}] \\ Q_{1/2}(\mathbf{r}) + (M_{\rm tot} - \frac{1}{2})(Q_{3/2}(\mathbf{r}) - Q_{1/2}(\mathbf{r})) & : M_{\rm tot} \in [\frac{1}{2}, \frac{3}{2}] \end{cases} . \quad (26)$$

Interestingly, $Q_{\rm ens}(\mathbf{r})$, and therefore also $\zeta_{\rm ens}(\mathbf{r})$, are *piecewise-linear* in $M_{\rm tot}$, for this scenario.

For completeness, consider the case of maximal ΔS_z, which is obtained, as we mentioned above, for $x = 1$ and $y = 0$. Then, the ensemble coefficients are $\gamma_{3/2} = \frac{1}{2} + \frac{1}{3}M_{\rm tot}$, $\gamma_{1/2} = \gamma_{-1/2} = 0$ and $\gamma_{-3/2} = \frac{1}{2} - \frac{1}{3}M_{\rm tot}$. Therefore, as we increase $M_{\rm tot}$, we obtain a linear transition between $|\Psi_{N_0,-3/2}\rangle\langle\Psi_{N_0,-3/2}|$ and $|\Psi_{N_0,3/2}\rangle\langle\Psi_{N_0,3/2}|$. The even moments of $\hat{S}_z$ equal $\langle \hat{S}_z^{2k} \rangle = S_0^{2k}$ and are constant, whereas the odd moments $\langle \hat{S}_z^{2k+1} \rangle = S_0^{2k} M_{\rm tot}$ are linear in $M_{\rm tot}$. The spin-densities $n^{\sigma}_{\rm ens}(\mathbf{r})$ and the spin distribution $Q_{\rm ens}(\mathbf{r})$ satisfy Eqs. (12) and (14), respectively, with $S_0 = \frac{3}{2}$.

In presence of a magnetic field $\mathbf{B}(\mathbf{r}) = B_0 f(\mathbf{r})\hat{z}$ in Case (d), the total energy becomes $\tilde{E}_{\rm ens}(N_0, M_{\rm tot}) = E(N_0) + \mu_{\rm B} B_0 M_{\rm tot} F_{N_0,3/2} + \mu_{\rm B} B_0 (F_{N_0,1/2} - F_{N_0,3/2}) y$. It can be minimized then with respect to y. As explained in the main text, we are looking then for the lowest and the highest possible values of y (see Fig. 3).

The lowest value, $y_{\rm min}$, can be expressed as

$$y_{\rm min}(M_{\rm tot}) = \begin{cases} -\frac{1}{2}M_{\rm tot} - \frac{3}{4} & : \ M_{\rm tot} \in [-\frac{3}{2}, -\frac{1}{2}] \\ \frac{1}{4}M_{\rm tot} - \frac{3}{8} & : \ M_{\rm tot} \in [-\frac{1}{2}, \frac{3}{2}] \end{cases} \quad (27)$$

(see Fig. 8). Due to the geometry of the $x - y$ domain in this Case, for each value of $y_{\rm min}$, there is *one* value of x:

$$x_1(M_{\rm tot}) := x(y_{\rm min}) = \begin{cases} -M_{\rm tot} - \frac{1}{2} & : \ M_{\rm tot} \in [-\frac{3}{2}, -\frac{1}{2}] \\ \frac{1}{2}M_{\rm tot} + \frac{1}{4} & : \ M_{\rm tot} \in [-\frac{1}{2}, \frac{3}{2}] \end{cases} \quad (28)$$

(see Fig. 9) As a result, the ensemble coefficients equal

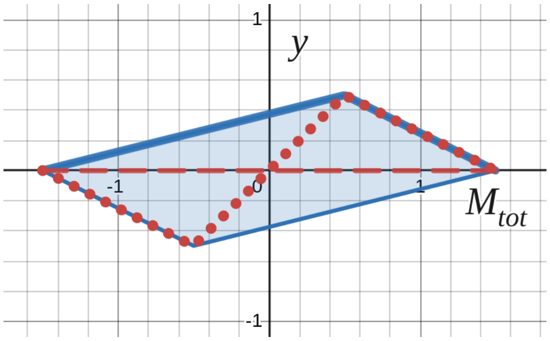

FIG. 8. Dependence of the minimal and the maximal values of y ($y_{\rm min}$, solid blue and $y_{\rm max}$, solid thick blue) on $M_{\rm tot}$, for $S_0 = \frac{3}{2}$. The functions $y_0(M_{\rm tot}) = y(x_{\rm min}(M_{\rm tot}))$ (dotted red) and $y_1(M_{\rm tot}) = y(x_{\rm max}(M_{\rm tot}))$ (dashed red) are shown for comparison.

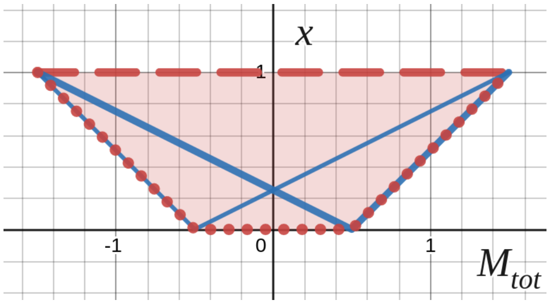

FIG. 9. Dependence of the x-values $x_1 = x(y_{\rm min})$ (solid blue) and $x_2 = x(y_{\rm max})$ (solid thick blue) on $M_{\rm tot}$, for $S_0 = \frac{3}{2}$. The functions $x_{\rm min}(M_{\rm tot})$ (dotted red) and $x_{\rm max}(M_{\rm tot})$ (dashed red) are shown for comparison.

$$
\begin{aligned}
\gamma_{3/2} &= \begin{cases} 0 & : \; M_{\rm tot} \in [-\frac{3}{2}, -\frac{1}{2}] \\ \frac{1}{2}M_{\rm tot} + \frac{1}{4} & : \; M_{\rm tot} \in [-\frac{1}{2}, \frac{3}{2}] \end{cases} \\
\gamma_{1/2} &= \begin{cases} 0 & : \; M_{\rm tot} \in [-\frac{3}{2}, -\frac{1}{2}] \\ 0 & : \; M_{\rm tot} \in [-\frac{1}{2}, \frac{3}{2}] \end{cases} \\
\gamma_{-1/2} &= \begin{cases} \frac{3}{2} + M_{\rm tot} & : \; M_{\rm tot} \in [-\frac{3}{2}, -\frac{1}{2}] \\ -\frac{1}{2}M_{\rm tot} + \frac{3}{4} & : \; M_{\rm tot} \in [-\frac{1}{2}, \frac{3}{2}] \end{cases} \\
\gamma_{-3/2} &= \begin{cases} -\frac{1}{2} - M_{\rm tot} & : \; M_{\rm tot} \in [-\frac{3}{2}, -\frac{1}{2}] \\ 0 & : \; M_{\rm tot} \in [-\frac{1}{2}, \frac{3}{2}] \end{cases}
\end{aligned}
\tag{29}
$$

(see Fig. 10 and compare to Fig. 7). Interestingly, for $M_{\rm tot} : -\frac{3}{2} \to -\frac{1}{2}$, we observe a linear transition from $\left|\Psi_{N_0,-3/2}\right\rangle\!\left\langle\Psi_{N_0,-3/2}\right|$ to $\left|\Psi_{N_0,-1/2}\right\rangle\!\left\langle\Psi_{N_0,-1/2}\right|$, as in the scenario of $(\Delta S_z)_{\rm min}$. But then, for $M_{\rm tot} : -\frac{1}{2} \to \frac{3}{2}$, we observe a transition from $\left|\Psi_{N_0,-1/2}\right\rangle\!\left\langle\Psi_{N_0,-1/2}\right|$ to $\left|\Psi_{N_0,3/2}\right\rangle\!\left\langle\Psi_{N_0,3/2}\right|$, skipping $\left|\Psi_{N_0,1/2}\right\rangle\!\left\langle\Psi_{N_0,1/2}\right|$. This results in ΔS_z which is higher than the minimal one:

$$
\Delta S_z(y_{\rm min}) = \begin{cases} \sqrt{(M_{\rm tot} + \frac{1}{2})(-M_{\rm tot} - \frac{3}{2})} & : M_{\rm tot} \in [-\frac{3}{2}, -\frac{1}{2}] \\ \sqrt{(M_{\rm tot} + \frac{1}{2})(\frac{3}{2} - M_{\rm tot})} & : M_{\rm tot} \in [-\frac{1}{2}, \frac{3}{2}] \end{cases}
\tag{30}
$$

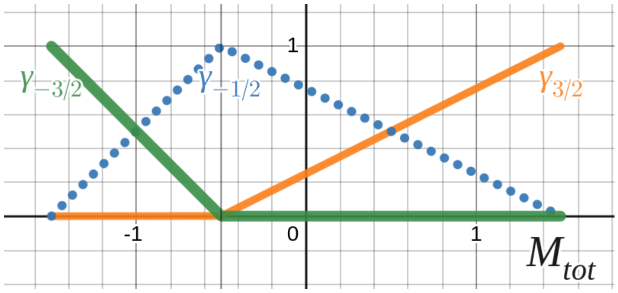

FIG. 10. Ensemble coefficients, $\gamma_{-3/2}$ (thick solid green), $\gamma_{-1/2}$ (dotted blue), and $\gamma_{3/2}$ (solid orange), versus $M_{\rm tot}$, for $S_0 = \frac{3}{2}$, for the scenario of $y_{\rm min}$. $\gamma_{1/2} = 0$ is not shown.

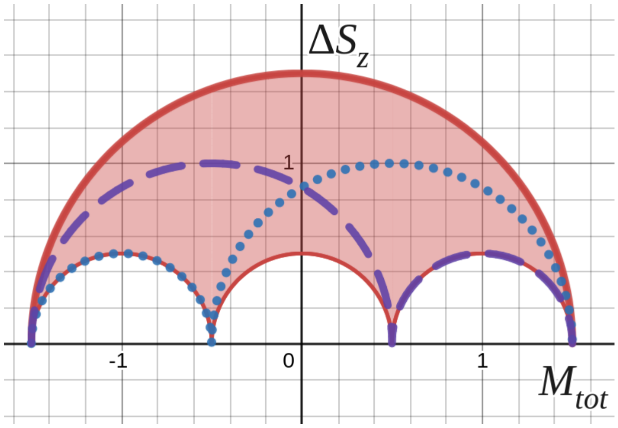

FIG. 11. Deviation ΔS_z versus $M_{\rm tot}$, for $S_0 = \frac{3}{2}$: for the scenario of a minimal y-value ($\Delta S_z(y_{\rm min})$, dotted blue) and for the scenario of a maximal y-value ($\Delta S_z(y_{\rm max})$, dashed purple). The minimal deviation, $(\Delta S_z)_{\rm min}$ (solid, thin red line) and the maximal deviation $(\Delta S_z)_{\rm max}$ (solid, thick red line) are brought for comparison.

(see Fig. 11) Similarly, for the highest possible y value, $y_{\rm max}$, we obtain the following results:

$$
y_{\rm max}(M_{\rm tot}) = \begin{cases} \frac{1}{4}M_{\rm tot} + \frac{3}{8} & : \; M_{\rm tot} \in [-\frac{3}{2}, \frac{1}{2}] \\ -\frac{1}{2}M_{\rm tot} + \frac{3}{4} & : \; M_{\rm tot} \in [\frac{1}{2}, \frac{3}{2}] \end{cases}
\tag{31}
$$

$$
x_2(M_{\rm tot}) := x(y_{\rm max}) = \begin{cases} \frac{1}{4} - \frac{1}{2}M_{\rm tot} & : \; M_{\rm tot} \in [-\frac{3}{2}, \frac{1}{2}] \\ -\frac{1}{2} + M_{\rm tot} & : \; M_{\rm tot} \in [\frac{1}{2}, \frac{3}{2}] \end{cases}
\tag{32}
$$

(see Figs. 8 and 9, respectively). The ensemble coeffi-

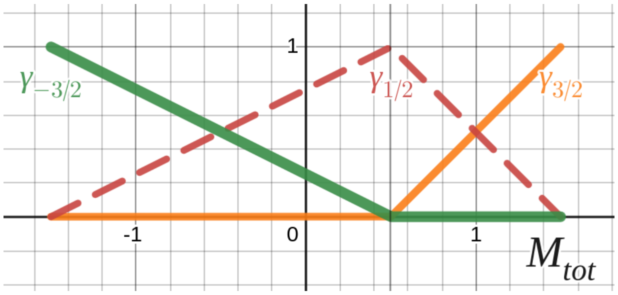

FIG. 12. Ensemble coefficients, $\gamma_{-3/2}$ (thick solid green), $\gamma_{1/2}$ (dashed red), and $\gamma_{3/2}$ (solid orange), versus $M_{\rm tot}$, for $S_0 = \frac{3}{2}$, for the scenario of $y_{\rm max}$. $\gamma_{-1/2} = 0$ is not shown.

cients equal

$$\gamma_{3/2} = \begin{cases} 0 & : M_{\rm tot} \in [-\frac{3}{2}, \frac{1}{2}] \\ M_{\rm tot} - \frac{1}{2} & : M_{\rm tot} \in [\frac{1}{2}, \frac{3}{2}] \end{cases}$$
$$\gamma_{1/2} = \begin{cases} \frac{3}{4} + \frac{1}{2}M_{\rm tot} & : M_{\rm tot} \in [-\frac{3}{2}, \frac{1}{2}] \\ \frac{3}{2} - M_{\rm tot} & : M_{\rm tot} \in [\frac{1}{2}, \frac{3}{2}] \end{cases} \tag{33}$$
$$\gamma_{-1/2} = \begin{cases} 0 & : M_{\rm tot} \in [-\frac{3}{2}, \frac{1}{2}] \\ 0 & : M_{\rm tot} \in [\frac{1}{2}, \frac{3}{2}] \end{cases}$$
$$\gamma_{-3/2} = \begin{cases} \frac{1}{4} - \frac{1}{2}M_{\rm tot} & : M_{\rm tot} \in [-\frac{3}{2}, \frac{1}{2}] \\ 0 & : M_{\rm tot} \in [\frac{1}{2}, \frac{3}{2}] \end{cases}$$

(see Fig. 12 and compare to Fig. 7). For $M_{\rm tot} : -\frac{3}{2} \to \frac{1}{2}$, we observe a linear transition from $|\Psi_{N_0,-3/2}\rangle\langle\Psi_{N_0,-3/2}|$ to $|\Psi_{N_0,1/2}\rangle\langle\Psi_{N_0,1/2}|$ (while skipping $|\Psi_{N_0,-1/2}\rangle\langle\Psi_{N_0,-1/2}|$), and then, for $M_{\rm tot} : \frac{1}{2} \to \frac{3}{2}$, from $|\Psi_{N_0,1/2}\rangle\langle\Psi_{N_0,1/2}|$ to $|\Psi_{N_0,3/2}\rangle\langle\Psi_{N_0,3/2}|$, as in the $(\Delta S_z)_{\rm min}$ scenario. This picture is complementary to the one we observed for $y_{\rm min}$: this time we skipped $|\Psi_{N_0,-1/2}\rangle\langle\Psi_{N_0,-1/2}|$. The result for ΔS_z with maximal y is:

$$\Delta S_z(y_{\rm max}) = \begin{cases} \sqrt{(\frac{1}{2} - M_{\rm tot})(M_{\rm tot} + \frac{3}{2})} & : M_{\rm tot} \in [-\frac{3}{2}, \frac{1}{2}] \\ \sqrt{(\frac{1}{2} - M_{\rm tot})(M_{\rm tot} - \frac{3}{2})} & : M_{\rm tot} \in [\frac{1}{2}, \frac{3}{2}] \end{cases} \tag{34}$$

(see Fig. 11). Therefore, in Case (d), introduction of a magnetic field and a subsequent minimization of the energy determines both x and y, and thus the ground state $\hat{\Gamma}$ unambiguously.

Finally, we discuss **Case (e)** of the main text: $N_{\rm tot} = N_0 + \alpha$, $\alpha \in [0, 1)$, $S_0 = \frac{1}{2}$ and $S_1 = 1$. This Case corresponds to adding an electron, e.g., to C^+ towards C, while varying $M_{\rm tot}$. In this Case we have five ensemble coefficients, with three constraints (Eq. (MT:9)). The coefficients can be expressed using two variables, x and y:

$$\begin{aligned} \gamma_{N_0,1/2} &= \frac{1-\alpha}{2} + y \\ \gamma_{N_0,-1/2} &= \frac{1-\alpha}{2} - y \\ \gamma_{N_0+1,1} &= \frac{\alpha - x}{2} + \frac{M_{\rm tot} - y}{2} \\ \gamma_{N_0+1,0} &= x \\ \gamma_{N_0+1,-1} &= \frac{\alpha - x}{2} - \frac{M_{\rm tot} - y}{2}. \end{aligned} \tag{35}$$

With these coefficients, we find that the total energy, $E(N_{\rm tot}, M_{\rm tot}) = (1-\alpha)E(N_0) + \alpha E(N_0+1)$, and $\langle\hat{S}_z\rangle = M_{\rm tot}$ are determined without ambiguity. In contrast, other quantities of interest depend on x or on y. The even moments of $\hat{S}_z$ equal $\langle\hat{S}_z^{2k}\rangle = S_0^{2k} + (S_1^{2k} - S_0^{2k})\alpha - S_1^{2k}x$, and are linear in x and independent of y, whereas the odd moments of $\hat{S}_z$ are $\langle\hat{S}_z^{2k+1}\rangle = S_0^{2k}y + S_1^{2k}(M_{\rm tot} - y)$ and are linear in y and independent of x. Similarly to Case (d), to determine the ensemble ground state, we can add two constraints, e.g., for $\langle\hat{S}_z^2\rangle$ and $\langle\hat{S}_z^3\rangle$.

The spin-densities are not uniquely determined. They can be expressed as

$$n^\sigma_{\rm ens}(\mathbf{r}) = \frac{1}{2}\left((1-\alpha)n_{N_0}(\mathbf{r}) + \alpha n_{N_0+1}(\mathbf{r})\right) + \\ + \frac{1}{2}\delta_\sigma\left[M_{\rm tot}Q_{N_0+1,1}(\mathbf{r}) + y\left(\frac{Q_{N_0,1/2}(\mathbf{r})}{1/2} - Q_{N_0+1,1}(\mathbf{r})\right)\right] \tag{36}$$

(cf. Eq. (18)). The spin-densities are independent of x and linear in y. It directly follows that the total density

$$n_{\rm ens}(N_{\rm tot}, M_{\rm tot}) = (1-\alpha)n_{N_0}(\mathbf{r}) + \alpha n_{N_0+1}(\mathbf{r}), \tag{37}$$

is piecewise-linear in α and unambiguously defined, as expected, while the spin distribution is given by

$$Q_{\rm ens}(\mathbf{r}) = (M_{\rm tot} - y)Q_{N_0+1,1}(\mathbf{r}) + y\frac{Q_{N_0,1/2}(\mathbf{r})}{1/2}. \tag{38}$$

It depends only on y, and can be determined by setting $\langle\hat{S}_z^3\rangle$, according to Sec. II.

As in previous Cases, we address the scenario of minimal ΔS_z. Here, $\Delta S_z = \sqrt{\frac{1}{4}(1+3\alpha) - x - M_{\rm tot}^2}$; a minimum is obtained for the highest possible x. The domain of x and y, which emerges from the requirement that all the ensemble coefficients are within $[0, 1]$, can be described by the inequalities: $0 \leqslant x \leqslant 1$, $|y| \leqslant \frac{1}{2}(1-\alpha)$ and $|y - M_{\rm tot}| \leqslant \alpha - x$. The $x - y$ domain is illustrated in Fig. 13.

In Case (e), the highest value of x is

$$x_{\rm max}(M_{\rm tot}) = \begin{cases} \frac{1+\alpha}{2} + M_{\rm tot} & : M_{\rm tot} \in [-\frac{1+\alpha}{2}, -\frac{1-\alpha}{2}] \\ \alpha & : M_{\rm tot} \in [-\frac{1-\alpha}{2}, \frac{1-\alpha}{2}] \\ \frac{1+\alpha}{2} - M_{\rm tot} & : M_{\rm tot} \in [\frac{1-\alpha}{2}, \frac{1+\alpha}{2}] \end{cases}. \tag{39}$$

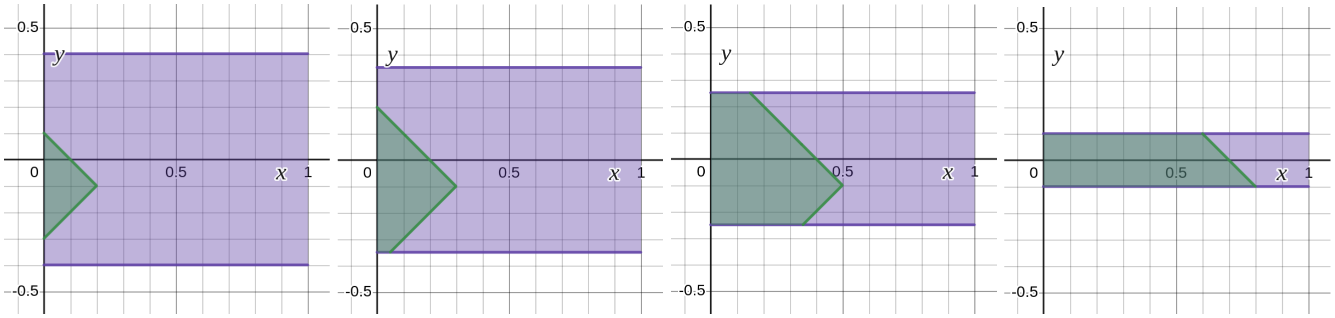

FIG. 13. The allowed domain of x and y, for Case (e) ($N_{\rm tot} = N_0 + \alpha$, $S_0 = \frac{1}{2}$, $S_1 = 1$), is the intersection of the green and purple areas. In all panels, $M_{\rm tot} = -0.1$, while $\alpha = 0.2$, 0.3, 0.5 and 0.8 (left to right). For an interactive plot, see: https://www.desmos.com/calculator/rtvugkfxap.

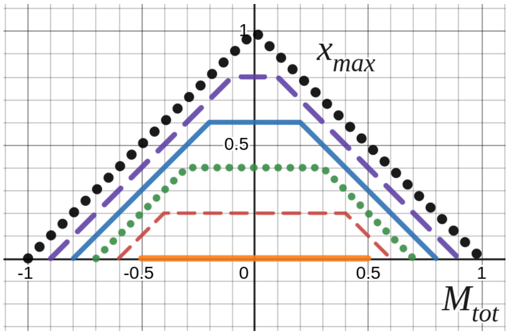

FIG. 14. Dependence of the maximal value of x ($x_{\rm max}$) on $M_{\rm tot}$, for different values of α: $\alpha = 0$ – solid orange, $\alpha = 0.2$ – dashed red, $\alpha = 0.4$ – dotted green, $\alpha = 0.6$ – solid blue, $\alpha = 0.8$ – dashed purple, $\alpha = 1$ – dotted black.

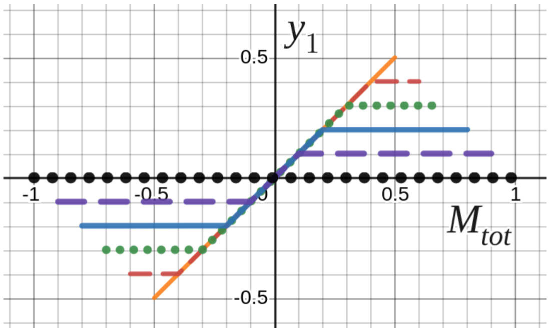

FIG. 15. Dependence of y_1 – the y-value that corresponds to the maximal value of x, on $M_{\rm tot}$, for different values of α. Color coding as in Fig. 14.

As in Case (d), also here for each value of $x_{\rm max}$ there is *one* corresponding value of y:

$$y_1(M_{\rm tot}) := y(x_{\rm max}) = \begin{cases} -\frac{1-\alpha}{2} & : \; M_{\rm tot} \in [-\frac{1+\alpha}{2}, -\frac{1-\alpha}{2}] \\ M_{\rm tot} & : \; M_{\rm tot} \in [-\frac{1-\alpha}{2}, \frac{1-\alpha}{2}] \\ \frac{1-\alpha}{2} & : \; M_{\rm tot} \in [\frac{1-\alpha}{2}, \frac{1+\alpha}{2}] \end{cases} \tag{40}$$

The dependence of $x_{\rm max}$ and y_1 on $M_{\rm tot}$, for different values of α, is depicted on Figs. 14 and 15, respectively. We note in passing that, in contrast to Case (d), *maximizing* ΔS_z does not uniquely determine the ground state. The lowest possible x, $x_{\rm min} = 0$, is always available, and corresponds to a range of y values.

The minimal value of ΔS_z is

$$(\Delta S_z)_{\rm min} = \begin{cases} \sqrt{\frac{\alpha-1}{4} - M_{\rm tot}(1+M_{\rm tot})} & : \; M_{\rm tot} \in [-\frac{1+\alpha}{2}, -\frac{1-\alpha}{2}] \\ \sqrt{\frac{1-\alpha}{4} - M_{\rm tot}^2} & : \; M_{\rm tot} \in [-\frac{1-\alpha}{2}, \frac{1-\alpha}{2}] \\ \sqrt{M_{\rm tot}(1-M_{\rm tot}) - \frac{1-\alpha}{4}} & : \; M_{\rm tot} \in [\frac{1-\alpha}{2}, \frac{1+\alpha}{2}] \end{cases} , \tag{41}$$

whereas the maximal value is $(\Delta S_z)_{\rm max} = \sqrt{\frac{1}{4}(1+3\alpha) - M_{\rm tot}^2}$; see Fig. 16. The particular cases for $\alpha = 0$ (Case (a)) and $\alpha = 1$ (Case (c)) are in agreement with derivations above.

For the scenario of minimal ΔS_z, the ensemble coefficients equal

$$\gamma_{N_0,1/2} = \begin{cases} 0 & : \; M_{\rm tot} \in [-\frac{1+\alpha}{2}, -\frac{1-\alpha}{2}] \\ \frac{1-\alpha}{2} + M_{\rm tot} & : \; M_{\rm tot} \in [-\frac{1-\alpha}{2}, \frac{1-\alpha}{2}] \\ 1-\alpha & : \; M_{\rm tot} \in [\frac{1-\alpha}{2}, \frac{1+\alpha}{2}] \end{cases}$$

$$\gamma_{N_0,-1/2} = \begin{cases} 1-\alpha & : \; M_{\rm tot} \in [-\frac{1+\alpha}{2}, -\frac{1-\alpha}{2}] \\ \frac{1-\alpha}{2} - M_{\rm tot} & : \; M_{\rm tot} \in [-\frac{1-\alpha}{2}, \frac{1-\alpha}{2}] \\ 0 & : \; M_{\rm tot} \in [\frac{1-\alpha}{2}, \frac{1+\alpha}{2}] \end{cases}$$

$$\gamma_{N_0+1,1} = \begin{cases} 0 & : \; M_{\rm tot} \in [-\frac{1+\alpha}{2}, -\frac{1-\alpha}{2}] \\ 0 & : \; M_{\rm tot} \in [-\frac{1-\alpha}{2}, \frac{1-\alpha}{2}] \\ M_{\rm tot} - \frac{1-\alpha}{2} & : \; M_{\rm tot} \in [\frac{1-\alpha}{2}, \frac{1+\alpha}{2}] \end{cases}$$

$$\gamma_{N_0+1,0} = \begin{cases} \frac{1+\alpha}{2} + M_{\rm tot} & : \; M_{\rm tot} \in [-\frac{1+\alpha}{2}, -\frac{1-\alpha}{2}] \\ \alpha & : \; M_{\rm tot} \in [-\frac{1-\alpha}{2}, \frac{1-\alpha}{2}] \\ \frac{1+\alpha}{2} - M_{\rm tot} & : \; M_{\rm tot} \in [\frac{1-\alpha}{2}, \frac{1+\alpha}{2}] \end{cases}$$

$$\gamma_{N_0+1,-1} = \begin{cases} -M_{\rm tot} - \frac{1-\alpha}{2} & : \; M_{\rm tot} \in [-\frac{1+\alpha}{2}, -\frac{1-\alpha}{2}] \\ 0 & : \; M_{\rm tot} \in [-\frac{1-\alpha}{2}, \frac{1-\alpha}{2}] \\ 0 & : \; M_{\rm tot} \in [\frac{1-\alpha}{2}, \frac{1+\alpha}{2}] \end{cases} . \tag{42}$$

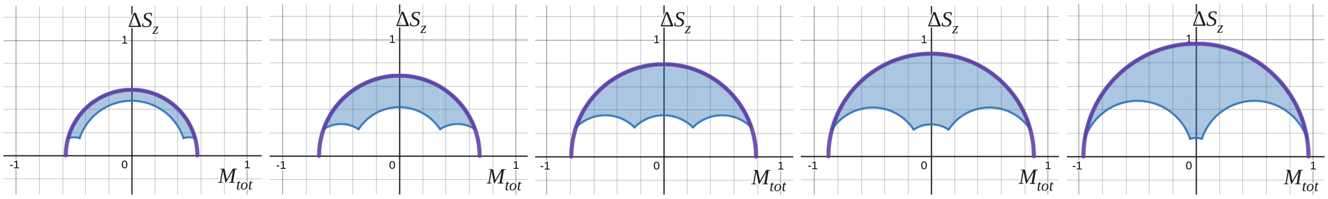

FIG. 16. Deviation ΔS_z versus $M_{\rm tot}$, for Case (e), for different values of α. From left to right: $\alpha = 0.1, 0.3, 0.5, 0.7, 0.9$. Minimal deviation, $(\Delta S_z)_{\rm min}$: solid blue line, maximal deviation, $(\Delta S_z)_{\rm max}$: thick solid purple line. For an interactive plot, see: https://www.desmos.com/calculator/msmk9qqtuu.

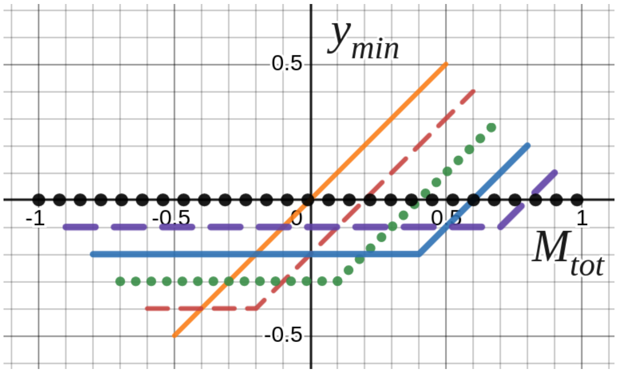

FIG. 17. Dependence of $y_{\rm min}$, the minimal value of y in Case (e), on $M_{\rm tot}$, for different values of α. Color coding as in Fig. 14.

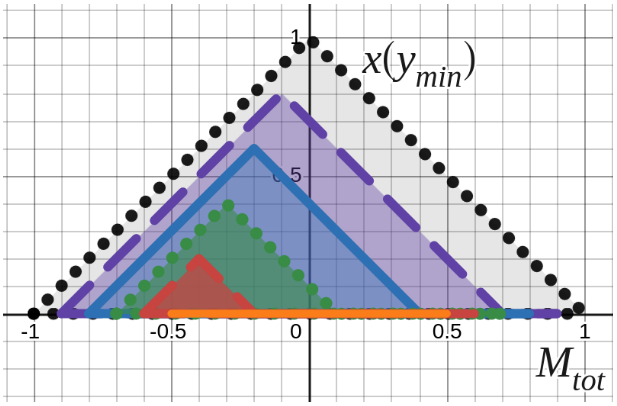

FIG. 18. Dependence of the allowed range of x-values, which correspond to the minimal y-value, in Case (e), on $M_{\rm tot}$. Color coding for different values of α as in Fig. 14. The range of allowed x-values is reflected by the shaded area on the plot.

For any value of $M_{\rm tot}$ we have three nonzero coefficients, at most.

Finally, the spin distribution for the case of minimal ΔS_z equals

$$Q_{\rm ens}(\mathbf{r}) = \begin{cases} (M_{\rm tot}+\frac{1-\alpha}{2})Q_{N_0+1,1}(\mathbf{r}) - \frac{1-\alpha}{2}\frac{Q_{N_0,1/2}(\mathbf{r})}{1/2} : M_{\rm tot}\in[-\frac{1+\alpha}{2}, -\frac{1-\alpha}{2}] \\ M_{\rm tot}\frac{Q_{N_0,1/2}(\mathbf{r})}{1/2} : M_{\rm tot}\in[-\frac{1-\alpha}{2}, \frac{1-\alpha}{2}] \\ (M_{\rm tot}-\frac{1-\alpha}{2})Q_{N_0+1,1}(\mathbf{r}) + \frac{1-\alpha}{2}\frac{Q_{N_0,1/2}(\mathbf{r})}{1/2} : M_{\rm tot}\in[\frac{1-\alpha}{2}, \frac{1+\alpha}{2}] \end{cases} . \quad (43)$$

Interestingly, also here, $Q_{\rm ens}(\mathbf{r})$, and therefore $\zeta_{\rm ens}(\mathbf{r})$, are piecewise-linear in both $M_{\rm tot}$ and in α.

In presence of a magnetic field $\mathbf{B}(\mathbf{r}) = B_0 f(\mathbf{r})\hat{z}$ in Case (e), the total energy becomes $\tilde{E}_{\rm ens}(N_{\rm tot}, M_{\rm tot}) = (1-\alpha)E(N_0) + \alpha E(N_0+1) + \mu_{\rm B}B_0 M_{\rm tot}F_{N_0+1,1} + \mu_{\rm B}B_0(F_{N_0,1/2} - F_{N_0+1,1})y$. Also here we are looking then for the lowest and the highest possible values of y.

Analyzing the $x-y$ domain of Case (e), we found that the lowest value, $y_{\rm min}$, can be expressed as

$$y_{\rm min}(M_{\rm tot}) = \begin{cases} -\frac{1-\alpha}{2} & : \ M_{\rm tot}\in[-\frac{1+\alpha}{2}, \frac{3\alpha-1}{2}] \\ M_{\rm tot}-\alpha & : \ M_{\rm tot}\in[\frac{3\alpha-1}{2}, \frac{1+\alpha}{2}] \end{cases} . \quad (44)$$

Importantly, in this Case, x is not determined uniquely: for $M_{\rm tot}\in[-\frac{1+\alpha}{2}, -\frac{1-\alpha}{2}]$, we find that $x\in[0, M_{\rm tot}+\frac{1+\alpha}{2}]$, for $M_{\rm tot}\in[-\frac{1-\alpha}{2}, \frac{3\alpha-1}{2}]$, we find that $x\in[0, -M_{\rm tot}+\frac{3\alpha-1}{2}]$, and for $M_{\rm tot}\in[\frac{3\alpha-1}{2}, \frac{1+\alpha}{2},]$, $x=0$. Figs. 17 and 18 show $y_{\rm min}$ and the range of x graphically. Therefore, while the magnetic field in this case can set the spin-densities, the odd spin moments, and other quantities, the ground state remains ambiguous (due to x).

References

(1) Weisstein, E. W., *CRC Concise Encyclopedia of Mathematics*, London: Chapman and Hall, 2003. Available on http://mathworld.wolfram.com.

(2) Harris, C. R. et al. Array Programming With NumPy. *Nature* **2020**, *585*, 357–362.
